# Metagenomic next-generation sequencing for tuberculosis diagnosis: enhanced performance and cost-effectiveness

**DOI:** 10.1128/spectrum.00970-26

**Published:** 2026-07-29

**Authors:** Xiaoli Zhou, Guo Wei, Tiange Song, Yanqiu Yu, Jianwei Chen, Juan Long, Xiaoyan Tao, Jie Zhang, Li Jiang

**Affiliations:** 1 Department of Laboratory Medicine, Sichuan Provincial People’s Hospital, School of Medicine, University of Electronic Science and Technology of China 12599, Chengdu, China; 2 Department of Clinical Research Center, Sichuan Provincial People’s Hospital, School of Medicine, University of Electronic Science and Technology of China 12599, Chengdu, China; 3 Department of Medical Information Centre, Sichuan Provincial People’s Hospital, School of Medicine, University of Electronic Science and Technology of China 12599, Chengdu, China; 4 Department of Accounting, Sichuan Provincial People’s Hospital, School of Medicine, University of Electronic Science and Technology of China 12599, Chengdu, China; Children's National Hospital, George Washington University, Washington, DC, USA; Maharishi Markandeshwar College of Medical Sciences and Research, Ambala, Haryana, India

**Keywords:** metagenomic next-generation sequencing (mNGS), *Mycobacterium tuberculosis *(MTB), tuberculosis (TB), PTB, EPTB, diagnostic performances, cost-effectiveness analysis

## Abstract

Metagenomic next-generation sequencing (mNGS) is a promising tool for diagnosing challenging infections like tuberculosis (TB). However, previous studies largely focused on case-specific application of mNGS in TB diagnosis. Thus, we conducted a retrospective observational study to first systematically evaluate the diagnostic performance and cost-effectiveness of mNGS for TB diagnosis. We retrieved a total of 16,776 results of the seven TB diagnostic assays, including mNGS, tuberculosis IgG antibody, TB interferon-γ release assay (TB-IGRA), TB-DNA, Xpert MTB/RIF (Xpert), culture, and acid-fast bacilli staining (AFS) from 3,757 participants with suspected TB infection at Sichuan Provincial People’s Hospital from September 2021 to July 2024. Diagnostic metrics were compared against a composite reference standard. Microbial composition and a cost-utility analysis were performed. Among seven TB assays studied, the World Health Organization (WHO)-recommended assays AFS, culture, and Xpert, as well as TB-IGRA, were requested most frequently for TB diagnosis, whereas mNGS ranked last. mNGS demonstrated the highest specificity (100%), accuracy (72.3%), and area under the curve (AUC) (0.795). Its sensitivity in bronchoalveolar lavage fluid and tissue was 71.0% and 72.7%, respectively. Sequential use of mNGS after initial WHO-recommended tests (Xpert/Culture/AFS) significantly improved diagnostic performance (sensitivity, 70.4%; AUC, 0.823). Microbial analysis associated *Candida albicans* with TB. Cost-utility analysis showed sequential mNGS became cost-effective at higher willingness-to-pay thresholds (>200,000 RMB per correct diagnosis). mNGS offers superior specificity for TB diagnosis. A sequential strategy applying mNGS to conventional-test-negative cases provides enhanced diagnostic performance and is cost-effective at higher healthcare investment values, supporting its utility for diagnostically challenging TB.

**IMPORTANCE:**

This study systematically assesses the diagnostic performance and cost utility of metagenomic next-generation sequencing (mNGS) for tuberculosis (TB) in a large real-world cohort of 3,757 suspected patients, comparing it against six conventional assays (tuberculosis IgG antibody, TB interferon-γ release assay, TB-DNA, Xpert, culture, and acid-fast bacilli staining). mNGS demonstrated the highest specificity (100%), accuracy (72.3%), and area under the curve (AUC) (0.795), with sensitivities of 71.0% in bronchoalveolar lavage fluid and 72.7% in tissue. Notably, sequential use of mNGS after the World Health Organization-recommended tests significantly improved sensitivity to 70.4% and AUC to 0.823. *Candida albicans* showed significant differences among the three groups. The sequential mNGS strategy was cost-effective compared with no mNGS, and its cost-effectiveness increased with a rising willingness-to-pay threshold. Overall, these results highlight mNGS as a valuable supplementary tool for challenging TB cases, especially when conventional tests are inconclusive, and provide strong evidence for integrating it into diagnostic algorithms to optimize clinical decision-making and resource allocation.

## INTRODUCTION

Tuberculosis (TB) is primarily caused by *Mycobacterium tuberculosis* (MTB), a member of the *Mycobacterium tuberculosis* complex, responsible for approximately 90% of TB cases worldwide (1). TB manifests as either active disease or latent infection. The disease predominantly affects the lungs (pulmonary TB, 70%–80% of cases) (2–4). Its common symptoms include chronic cough (occasionally with hemoptysis), fever, fatigue, night sweats, anorexia, and weight loss—clinical features that often overlap with infections caused by non-tuberculous mycobacteria, complicating differential diagnosis (2, 5, 6).

In 2014, the World Health Assembly endorsed the World Health Organization (WHO) End TB Strategy, which aimed to reduce the global TB incidence by 80% by 2030 (3). However, between 2020 and 2023, global TB incidence increased by 4.6%, with an estimated 10.8 million new cases reported in 2023. China contributed significantly to this burden, accounting for 6.8% of global cases (2, 3). In 2023, TB re-emerged as the world’s leading cause of death from an infectious disease, surpassing COVID-19. It remained the foremost cause of mortality among people living with HIV and a major driver of antimicrobial resistance-related deaths. Therefore, accurate and early detection of TB is critical to curbing transmission, preventing the emergence of drug-resistant TB, and guiding timely treatment interventions.

Current methods of MTB detection in clinical laboratories can be categorized into microbiological, immunological, and molecular techniques. Among them, MTB culture is the gold standard for TB diagnosis and also provides comprehensive drug susceptibility profiling at low cost, but it is constrained by prolonged turnaround time and modest sensitivity (7, 8). Acid-fast staining (AFS), though rapid and economical, offers limited sensitivity and specificity, particularly in paucibacillary populations such as children or people living with HIV (9–11). In addition, the immunological assays, such as the tuberculin skin test (TST), interferon-gamma release assays (IGRAs), and MTB-specific IgG (TB-IgG) assay, are limited by their dependence on host immune competence, which can lead to variable sensitivity and specificity. Moreover, TST is invasive and requires skilled administration (12–15). Molecular methods like the MTB-DNA PCR assay and the GeneXpert MTB/RIF assay (Xpert) allow rapid detection and identification of rifampicin resistance, yet their accuracy is influenced by sample type and patient population (9, 16–19). Additionally, the 2018 WHO Essential Diagnostics List included loop-mediated isothermal amplification (TB-LAMP) for pulmonary tuberculosis in primary care. Its advantages include simplicity, rapid results, and the ability to replace sputum smear microscopy as an initial test. However, the recommendation is conditional and based on very low-quality evidence, highlighting the need for further validation (20, 21). Consequently, there remains a pressing need for novel diagnostic strategies that are both rapid and accurate to improve MTB diagnosis and clinical management.

Metagenomic next-generation sequencing (mNGS) was first introduced into clinical practice in 2008. As a powerful non-targeted pathogen detection assay, it is capable of simultaneously identifying bacteria, viruses, fungi, and parasites from a wide range of clinical samples, including blood, bronchoalveolar lavage fluid, cerebrospinal fluid, and tissue (22–30). A few groups reported that mNGS significantly improves MTB detection, particularly in immunocompromised patients, thereby supporting earlier intervention and better clinical outcomes (31–33).

However, previous studies have largely focused on case-specific applications of mNGS, and there has barely been a comprehensive evaluation of its diagnostic utility and cost-effectiveness for TB diagnosis (34–36). Thus, we conducted a large retrospective study to systematically evaluate not only the diagnostic performance of mNGS for TB, with comparison to conventional methods, including serological (TB-IgG), immunological (TB-IGRA), molecular (TB-DNA, Xpert), and microbiological methods (culture, AFS), but also its utility in extrapulmonary TB (EPTB) and characterization of co-infecting pathogens with MTB. Most importantly, we have first evaluated the cost-effectiveness of a sequential mNGS following the WHO-recommended conventional methods for TB patient care.

## MATERIALS AND METHODS

### Study design and population

This retrospective observational study enrolled 3,757 inpatients clinically suspected of tuberculosis infection based on symptoms including fever, night sweats, and weight loss, who underwent at least one TB-related assay at Sichuan Provincial People’s Hospital between September 2021 and July 2024. According to the China Clinical Treatment Guide for Tuberculosis (2017 edition), all participants were classified into non-TB, pulmonary TB (PTB), and extrapulmonary TB, with the categorization of those diagnosed with both PTB and EPTB into the PTB group (2). Seven TB diagnostic assays were provided by the hospital central laboratory, including mNGS, TB-IgG, TB-IGRA, TB-DNA, Xpert MTB/RIF, culture, and acid-fast staining. Although WHO has not recommended the use of serological antibody tests for active TB diagnosis, TB-IgG assays remain in clinical use in some resource-limited regions of China. Therefore, we included this assay to evaluate its real-world performance. The total medical care costs of each participant during hospitalization were retrieved from the hospital information system.

### Composite reference standard and adjudication of discordant results

The final clinical diagnosis recorded in the medical records served as the composite reference standard. The diagnosis was based on the Chinese Clinical Treatment Guide for Tuberculosis (2017 edition) and WHO recommendations, integrating microbiological evidence (positive culture or Xpert MTB/RIF), radiological findings (chest CT), clinical symptoms (cough ≥2 weeks, fever, night sweats, and weight loss), treatment response to anti-TB therapy, and exclusion of alternative diagnoses. Importantly, mNGS was performed retrospectively and was not available to the treating physicians at the time of diagnosis, ensuring independence of the reference standard from the index test. For discordant results (e.g., negative microbiological tests but strong radiological and clinical suspicion), the final diagnosis was determined by two licensed physicians (JL and JZ) who were blinded to the mNGS results; disagreements were resolved by consensus or by consultation with a third senior clinician (XYT). Patients with insufficient data for definitive classification were excluded from the primary analysis.

### Six conventional laboratory assays for TB diagnosis

In this study, six assays were routinely conducted at the central laboratory for TB diagnosis, including immunological, molecular, and microbiological methods. Serum/plasma samples were tested for TB-specific IgG antibodies using a commercial ELISA kit (Wantai Biology, Beijing, China) as a serological auxiliary assay. T-cell immune response to MTB was assessed via a chemiluminescence-based interferon-gamma release assay (Wantai Kairui, Xiamen, China), quantifying IFN-γ production after stimulation with ESAT-6 and CFP-10 antigens. A PCR-fluorescent probe assay (DaAn Gene, China) was used to detect MTB complex DNA (TB-DNA). The Xpert MTB/RIF assay (Cepheid, Sweden) was applied to various sample types, including sputum, bronchoalveolar lavage fluid (BALF), urine, and cerebrospinal fluid (CSF) following the manufacturer’s instructions.

Microbiological confirmation was conducted using culture-based methods: the automated BD BACTEC MGIT 960 system (Becton, Dickinson, USA) and Lowenstein-Jensen solid medium (BaSO, Zhuhai, China), with incubation for up to 42 days. Sterile fluid samples (e.g., urine, pus, ascites, pleural fluid, BALF, and CSF) were centrifuged at 3,000 rpm for 15 min to concentrate specimens; sediments were subjected to AFS using commercial reagents (BA4091, BaSO, Zhuhai, China).

### mNGS for MTB detection

The central laboratory used a commercial mNGS kit to detect MTB from diverse clinical samples (CSF, BALF, sputum, pleural fluid, tissue, and ascites) by following the manufacturer’s protocol. Before DNA extraction with TIANamp Micro DNA Kit (DP316, TIANGEN BIOTECH), each sample underwent cell lysis involving lysozyme treatment and mechanical homogenization with glass beads (6 m/s, 45 s, two cycles), except blood, which proceeded directly to DNA extraction due to high free DNA/RNA content. Then, DNA libraries were prepared with the NEBNext Ultra II DNA Library Prep Kit (New England Biolabs Inc.) and loaded into the Illumina NextSeq 550 DX platform (75 bp single-end reads, ~20 million reads per sample) for sequencing.

Bioinformatic analysis included checking for quality control, removal of human sequences (mapped to GRCh38) via Burrows-Wheeler Alignment, and alignment to the NCBI database using SNAP for taxonomic annotation, genome coverage/depth calculation, and abundance analysis.

To minimize contamination, strict physical separation of pre- and post-PCR areas with a unidirectional workflow was enforced. Each sequencing batch included a negative control (extraction blank) and a positive control (mock community). Reads belonging to a predefined list of common environmental background genera were removed from the final report. If contamination was suspected by the reviewing physician based on control results or abnormal read patterns, the sample was retested.

### Performance evaluation of TB diagnostic assays

Diagnostic accuracy metrics, including sensitivity, specificity, accuracy, positive predictive value (PPV), negative predictive value (NPV), and receiver operating characteristic (ROC) curve analysis, were calculated for mNGS, TB-IgG, TB-IGRA, TB-DNA, Xpert, culture, AFS, and two combinations of the WHO-recommended testing (Xpert, culture, and AFS) with or without sequential mNGS using parallel testing, with clinical diagnosis as the reference standard. Confidence intervals (CIs) for sensitivity, specificity, accuracy, PPV, NPV, and ROC were calculated using the Wilson score method in R using the “PropCIs” package. The McNemar test for paired fourfold tables was used to compare mNGS with the other six methods.

### Cost-effectiveness analysis of the WHO-recommended assays with or without sequential mNGS

Two diagnostic strategies were compared: the WHO-recommended combination testing (Xpert, culture, and AFS) versus sequential mNGS. The reference standard was clinically confirmed tuberculosis. A correct diagnosis was defined as agreement between the laboratory result (Xpert + culture + AFS or sequential mNGS) and the reference standard. The total healthcare cost during hospitalization of each patient (RMB) was modeled using a generalized linear model (GLM) with Gamma family and log link, adjusting for demographic (age, sex, and smoking), comorbidities (diabetes, tumor, hypertension, and immunosuppression), surgery status, and inflammatory marker categories. Cluster-robust standard errors were used at the inpatient-ID level.

Incremental cost-effectiveness ratios were estimated as the difference in mean cost divided by the difference in laboratory diagnostic accuracy between strategies. Clustered nonparametric bootstrapping (1,000 replications) generated joint distributions of incremental cost and effectiveness. From these, net monetary benefits were calculated over a range of willingness-to-pay (WTP) thresholds (0–400,000 RMB) to construct the cost-effectiveness acceptability curve (CEAC). All analyses were conducted in Stata 18 (StataCorp, College Station, TX, USA).

### Statistical analysis

Statistical analyses were conducted using GraphPad Prism (v6.01), SPSS (v25, IBM Corp), and R (v4.1.2). Continuous variables are expressed as median values; categorical variables are presented as frequencies or percentages (%). A two-tailed *P*-value < 0.05 was considered statistically significant. Subsequently, all heat maps were used to visualize the pathogen distribution or test results by the R package “pheatmap.” The Spearman correlation analysis was conducted by R package “corrplot.” The alpha diversity index was calculated based on Shannon and Simpson indices. Beta-diversity was visualized using principal coordinate analysis (PCoA), and an ANOSIM test was performed in R using the “Vegan” package. The stacked bar plot of the community composition was visualized in R using the “ggplot2” package. Linear discriminant analysis (LDA) effect size (LEfSe) was utilized at https://cloud.oebiotech.com to identify significantly different species among the groups, with thresholds of log10 LDA Score ≥2 and *P* value ≤ 0.05.

## RESULTS

### Characteristics of study participants

A total of 3,757 inpatients with suspected TB infection at Sichuan Provincial People’s Hospital between September 2021 and July 2024 were recruited in this study (Table 1). They all underwent at least one of seven TB diagnostic assays, including mNGS, TB-IgG, TB-IGRA, TB-DNA, Xpert, Culture, and AFS, routinely provided by the hospital central laboratory. According to the WHO diagnostic guideline for TB, the participants were classified into three groups: PTB (*n* = 1,387, 36.9%), EPTB (*n* = 900, 24.0%), and non-TB (*n* = 1,470, 39.1%) groups. There were significant differences in gender, diabetes, tumors, hypertension, procalcitonin (PCT), high-sensitivity C-reactive protein (hsCRP), immunosuppression, lymphocyte counts, lymphocyte percentage, IFN-γ, erythrocyte sedimentation rate (ESR), CD4+ T cells, and CD8+ T cells among the three groups (all *P* < 0.05) (Table 1). Spearman correlation analyses also showed significant positive correlations between TB infection and male, PCT, hsCRP, and ESR and significant negative correlations between TB infection and the following variables: age, hypertension, and tumors (Fig. S1).

**TABLE 1:** General demographic and clinical characteristics of all enrolled patients in three groups^*a*^

	Total (*n* = 3,757)	PTB group (*n* = 1,387)	EPTB group (*n* = 900)	Non-TB group (*n* = 1,470)	*P*
Sex, *n* (%)					
Female	1,552	375 (24.2%)	480 (30.9%)	697 (44.9%)	<0.001
Male	2,205	525 (23.8%)	907 (41.1%)	773 (35.1%)	
Smoking, *n* (%)					
No	3,731	895 (24.0%)	1,375 (36.9%)	1,461 (39.2%)	0.61
Yes	26	5 (19.2%)	12 (46.2%)	9 (34.6%)	
Diabetes, *n* (%)					
No	3,128	782 (25.0%)	1,115 (35.6%)	1,231 (39.4%)	<0.001
Yes	629	118 (18.8%)	272 (43.2%)	239 (38.0%)	
Tumors, *n* (%)					
No	3,182	833 (26.2%)	1,211 (38.1%)	1,138 (35.8%)	<0.001
Yes	575	67 (11.7%)	176 (30.6%)	332 (57.7%)	
Hypertension, *n* (%)					
No	2,752	687 (25.0%)	1,053 (38.3%)	1,012 (36.8%)	<0.001
Yes	1,005	213 (21.2%)	334 (33.2%)	458 (45.6%)	
Immunosuppression, *n* (%)					
No	3,369	841 (25.0%)	1,237 (36.7%)	1,291 (38.3%)	<0.001
Yes	388	59 (15.2%)	150 (38.7%)	179 (46.1%)	
Laboratory examination, median value					
PCT (ng/mL)	0.07 (0.05–0.18)	0.08 (0.05–0.21)	0.09 (0.05–0.23)	0.06 (0.04–0.12)	<0.001
hsCRP (mg/dL)	14.3 (3.4–46.9)	21.6 (6.2–49.7)	19.5 (4.0–61.6)	8.2 (2.0–28.2)	<0.001
Lymphocytes (×10^9^/L)	1.12 (0.79–1.49)	0.98 (0.68–1.35)	1.05 (0.70–1.43)	1.27 (0.93–1.64)	<0.001
Lymphocyte percentage (%)	19.0 (13.1–26.4)	17.7 (12.9–25.0)	17.5 (11.1–24.9)	21.3 (14.9–28.1)	<0.001
IFN-γ (pg/mL)	118.8 (3.7–897.5)	196.1 (7.4–928.0)	113.1 (4.4–513.4)	93.2 (2.5–1,659.4)	0.032
ESR (mm/h)	52.0 (26.0–82.0)	57.0 (32.0–84.0)	56.0 (25.0–87.0)	46.0 (24.0–78.5)	<0.001
CD4+ T cell (μL)	393.0 (224.0–588.0)	288.0 (161.0–481.5)	305.0 (179.0–477.0)	473.0 (301.0–656.0)	<0.001
CD8+ T cell (μL)	260.0 (163.0–404.0)	211.0 (127.0–320.0)	218.5 (136.5–361.0)	295.0 (195.0–440.0)	<0.001

^*a*^ PCT, procalcitonin; hsCRP, high-sensitivity C-reactive protein. Data summarized by median interquartile range and compared by a Kruskal-Wallis test.

**Fig 1:**
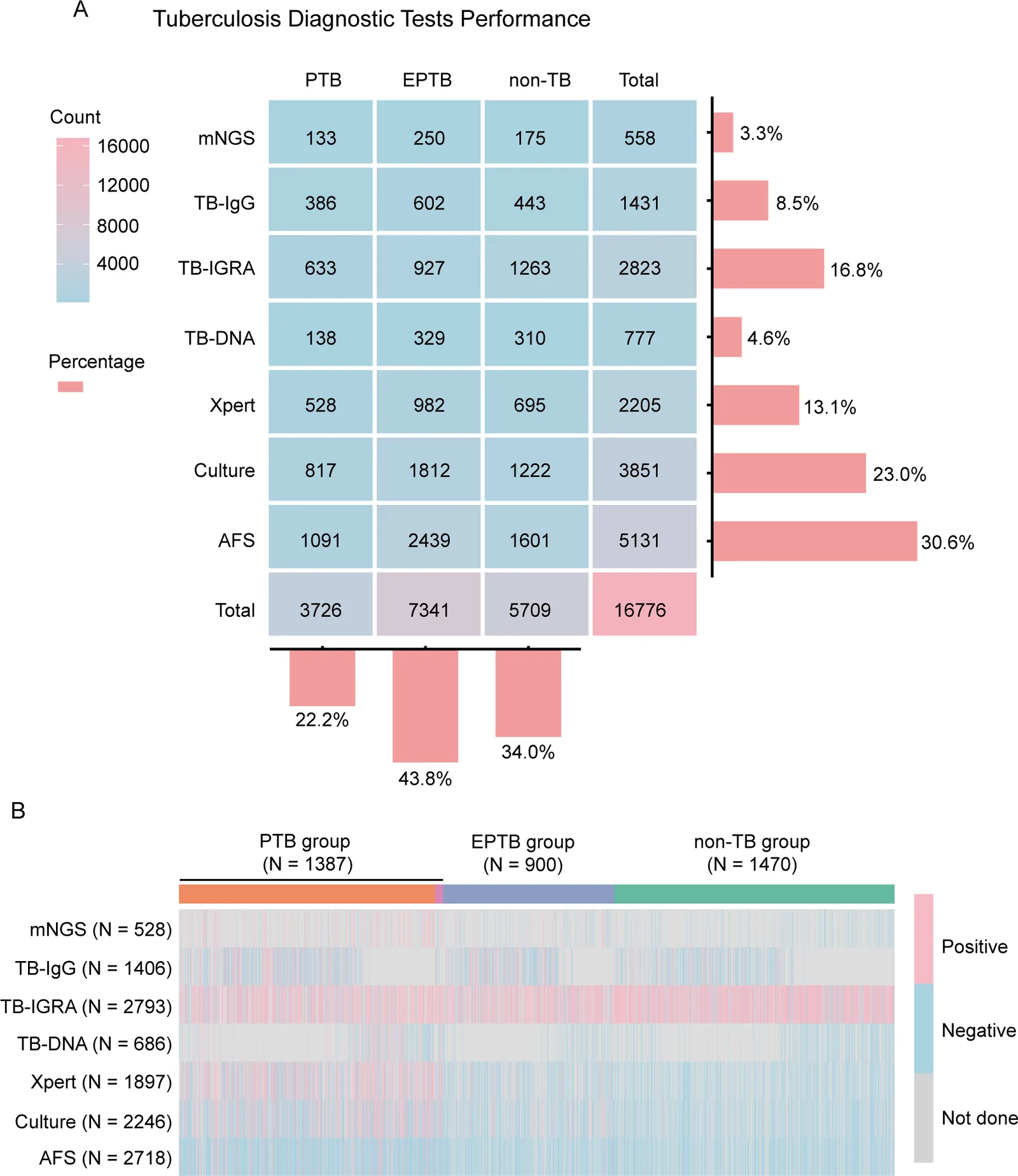
Summary of the request volumes and the results of the seven TB diagnostic assays for the participants. (**A**) Request volumes and percentages of the seven assays among the three groups. (**B**) Distribution of the seven assay results among the three groups.

In our study, a total of 16,776 requests of the seven TB diagnostic assays by physicians were carried out for the 3,757 patients. We analyzed the request volumes of the different assays and ranked them in descending order as follows: AFS (*n* = 5,131, 30.6%), culture (*n* = 3,851, 23.0%), TB-IGRA (*n* = 2,823, 16.8%), Xpert (*n* = 2,205, 13.1%), TB-IgG (*n* = 1,431, 8.5%), TB-DNA (*n* = 777, 4.6%), and mNGS (*n* = 558, 3.3%) (Fig. 1A; Data set S1 ). Our results revealed that AFS, culture, TB-IGRA, and Xpert were the top four assays routinely requested for TB diagnosis by physicians, whereas mNGS ranked last. Among the three groups, the EPTB group received nearly half of TB diagnostic assay requests (*n* = 7,341, 43.8%), which was substantially higher than both PTB (*n* = 3,726, 22.2%) and non-TB (*n* = 5,709, 34.0%) groups (Fig. 1A; Data set S1). In addition, the positive rates of the seven assays were found to be substantially different, with TB-IGRA at the highest positive rate of 80.7%, and AFS at the lowest rate of 6.8% among them (Fig. 1B; Table 2).

**TABLE 2:** The results of mNGS, TB-IgG, TB-IGRA, TB-DNA, Xpert, culture, and AFS

Methods	Sample size (*n*)	Positive (*n*)	Negative (*n*)	Positive rate (%)
mNGS	528	210	318	39.8
TB-IgG	1,406	446	960	31.7
TB-IGRA	2,793	2,255	538	80.7
TB-DNA	686	120	566	17.5
Xpert	1,897	566	1,331	29.8
Culture	2,246	497	1,749	22.1
AFS	2,718	186	2,532	6.8

### Diagnostic performances of mNGS and other conventional TB assays

**Fig 2:**
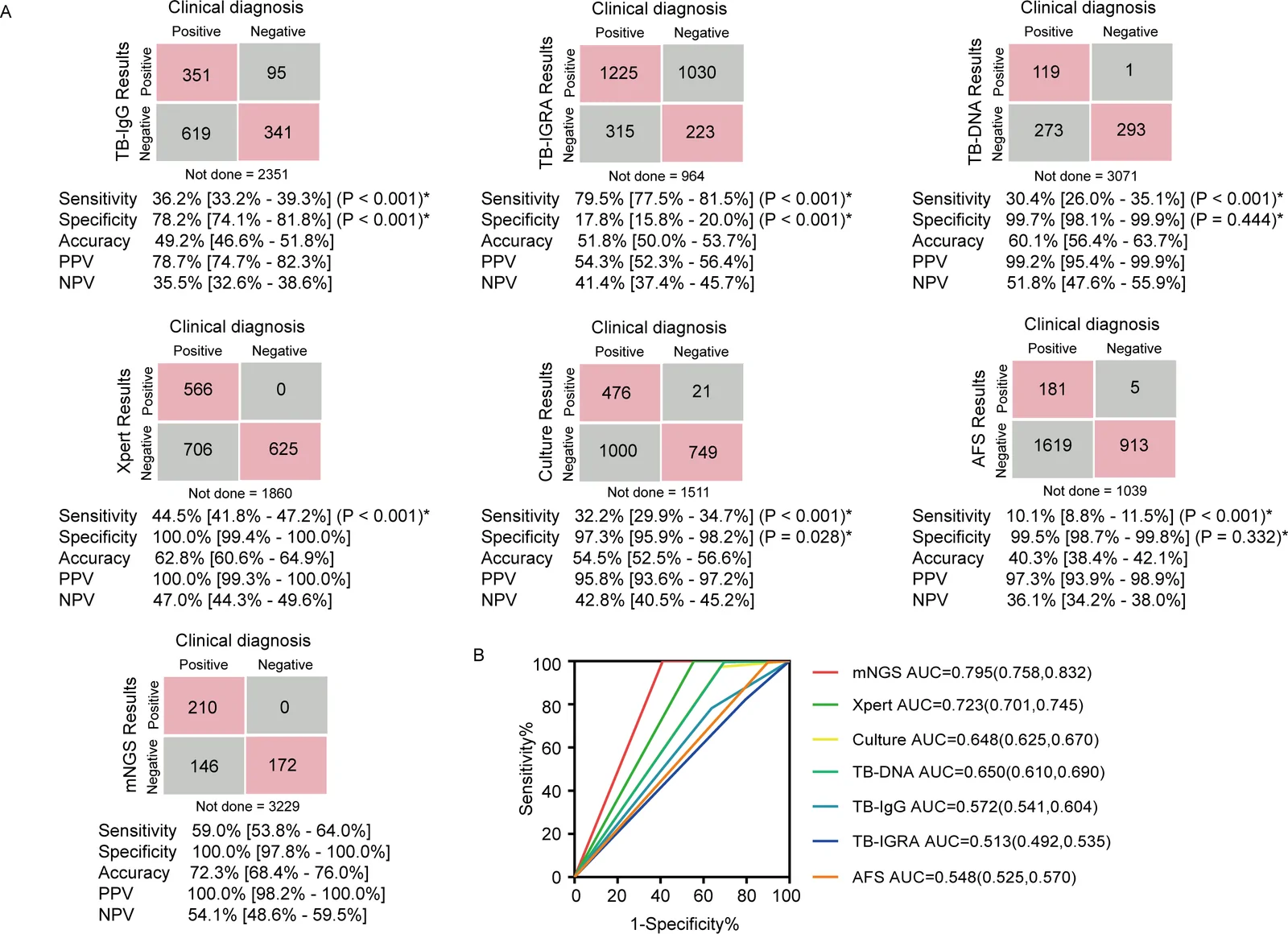
Diagnostic performances of mNGS and six conventional TB assays. (**A**) Contingency tables (2 × 2) showing the comparative performance of mNGS testing compared to other six TB diagnostic assays. The *P* value is based on comparison between mNGS and a conventional testing modality using the two-sided McNemar’s test. NPV, negative predictive value; PPV, positive predictive value. *Comparison with mNGS using McNemar’s test. (**B**) ROC curve of mNGS, TB-IgG, TB-IGRA, TB-DNA, Xpert, culture, and AFS individually for discrimination between TB and non-TB infection by negative or positive.

Based on 16,776 testing results retrieved from 3,757 participants in our study, we first evaluated the sensitivity, specificity, and accuracy of mNGS and the other six conventional TB diagnostic assays. It revealed that the sensitivities of the seven assays, in descending order, were as follows: TB-IGRA (79.5%), mNGS (59.0%), Xpert (44.5%), culture (32.2%), TB-DNA (30.4%), TB-IgG (26.2%), and AFS (10.1%) (Fig. 2A; Data set S2). Among the seven assays, TB-IGRA and AFS showed the highest and lowest sensitivities, respectively, which is consistent with their positive rates. In addition, both mNGS and Xpert exhibited excellent specificities of 100%, followed by TB-DNA (99.2%), AFS (97.5%), culture (97.3%), TB-IgG (78.2%), and TB-IGRA (17.8%) (Fig. 2A). Notably, mNGS displayed the best accuracy at 72.3% compared to Xpert at 62.8%, TB-DNA at 60.1%, culture at 54.5%, TB-IGRA at 51.8%, TB-IgG at 49.2%, and AFS at 40.3% (Fig. 2A). Furthermore, we analyzed the PPV and NPV of the seven assays with the same set of data. Both mNGS and Xpert also showed the best PPV at 100%, followed by TB-DNA at 99.7%, AFS at 97.3%, culture at 95.8%, TB-IgG at 78.7%, and TB-IGRA at 54.3%, with their trends being consistent with their specificities (Fig. 2A). The seven assays exhibited relatively low NPVs, with TB-IgG at the lowest of 35.5% and mNGS at the highest of 54.1% (Fig. 2A). To assess the discriminative power of the seven assays, we performed ROC curve analysis, and the areas under the curves (AUCs), in descending order, were as follows: mNGS (0.795; 95% CI, 0.758–0.832), Xpert (0.732; 95% CI, 0.701–0.745), culture (0.648; 95% CI, 0.625–0.670), TB-DNA (0.650; 95% CI, 0.610–0.690), TB-IgG (0.572; 95% CI, 0.541–0.604), TB-IGRA (0.513; 95% CI, 0.492–0.535), and AFS (0.548; 95% CI, 0.525–0.570) (Fig. 2B). Thus, our study revealed that mNGS was the best-performing assay overall in specificity, PPV, accuracy, and discriminative ability (AUC) over the other TB diagnostic assays.

**Fig 3:**
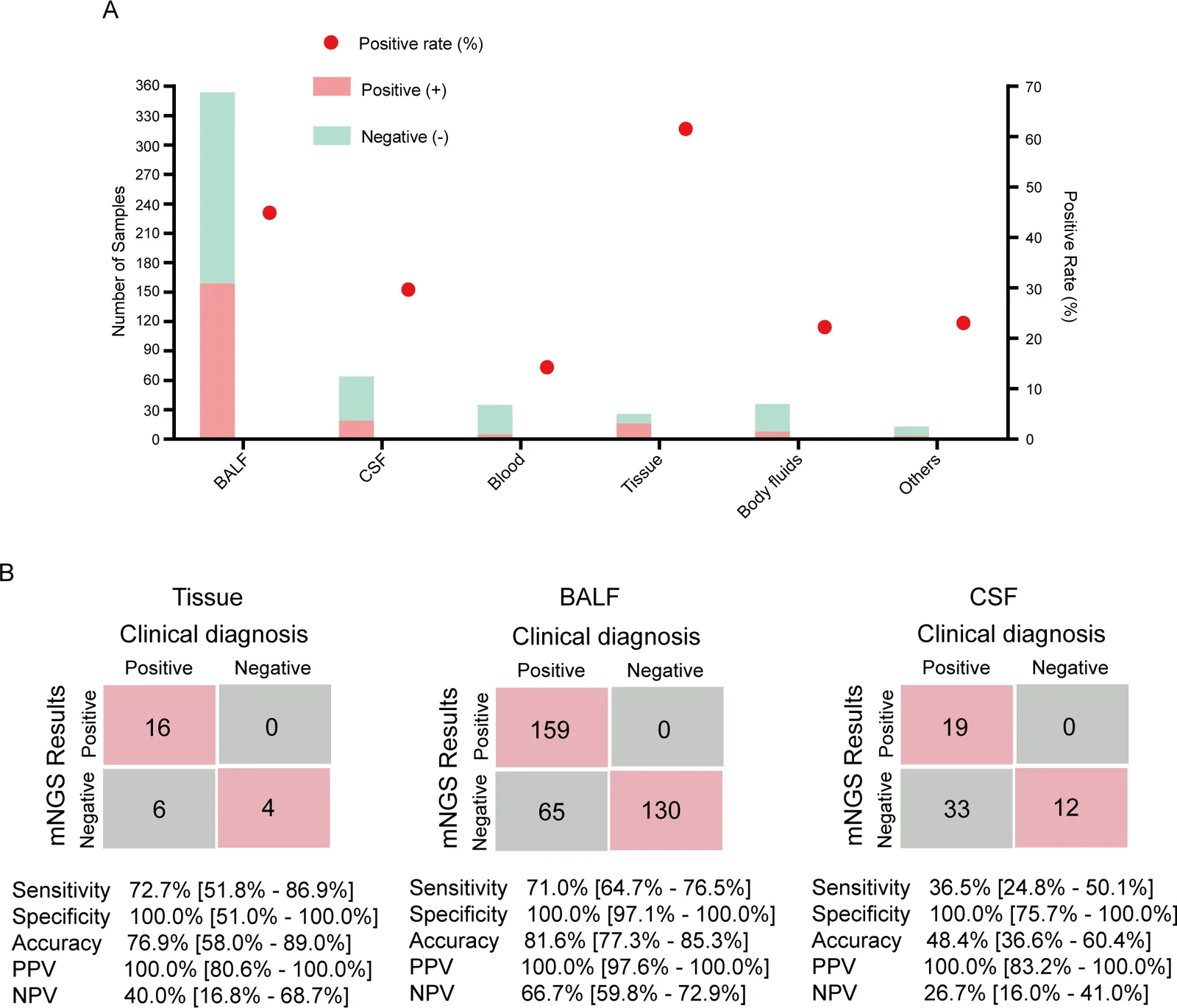
Diagnostic performance of mNGS in different sample types. (**A**) mNGS MTB-positive results of different sample types. (**B**) Contingency tables (2 × 2) showing the diagnostic performance of mNGS for MTB detection compared to the comprehensive diagnostic standard in tissue, BALF, and CSF samples.

Noticeably, mNGS is the only method capable of detecting MTB from multiple types of specimens, including blood, tissue, BALF, CSF, and body fluids. In our study, the top three specimen types used were tissue (61.54%), BALF (44.92%), and CSF (29.69%) (Fig. 3A; Dataset S3). We compared the diagnostic performances of mNGS in all specimen types (Table S1) and showed the top three types in Fig. 3. The specificities and PPVs were all 100% overall, while both tissue (72.7%) and BALF (71.0%) had superior sensitivities compared to CSF (36.5%). Especially, mNGS possessed the highest accuracy (81.6%), NPV (66.7%), and AUC (0.855; 95% CI, 0.816–0.894) in BALF among all types (Fig. 3B; Fig. S2; Table S1), indicating BALF is the best specimen type for TB diagnosis with mNGS.

**Fig 4:**
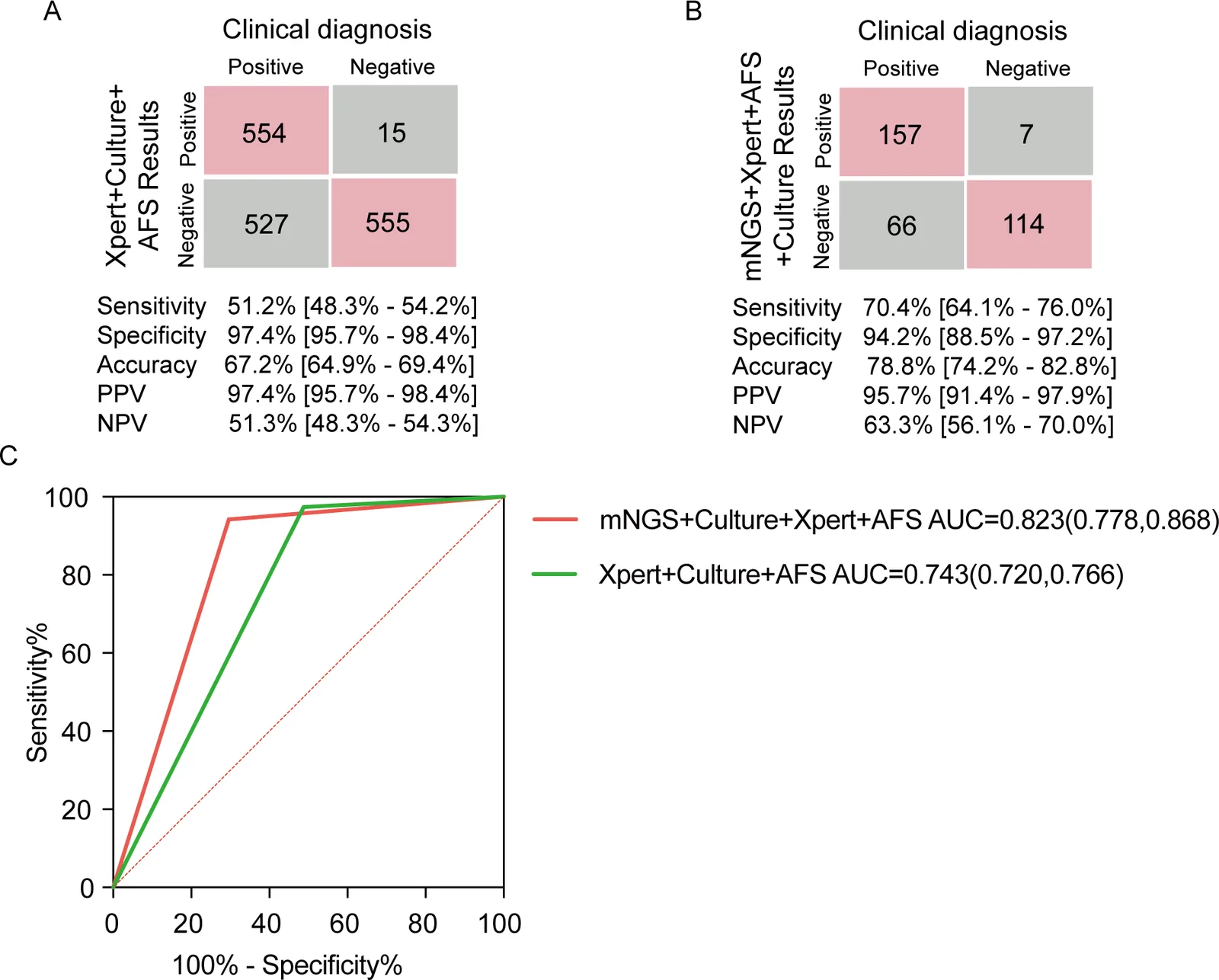
Combined diagnostic performance of mNGS with Xpert, culture, and AFS. (**A**) Diagnostic performance of Xpert combined with culture and AFS for MTB detection compared to the comprehensive diagnostic standard. (**B**) Combined diagnostic performance of mNGS combined with Xpert, culture, and AFS, evaluated against the comprehensive diagnostic standard for TB detection. Five indices with 95% CI are provided below each matrix. (**C**) ROC curve of mNGS combined with Xpert, culture, and AFS (mNGS＋Xpert＋culture＋AFS) individually for discrimination between TB and non-TB infection by negative or positive.

It is well known that WHO recommends Xpert, culture, and AFS as conventional methods used for TB diagnosis (2). To test whether mNGS could improve the diagnostic performance after these recommended assays, we evaluated the sensitivity, specificity, accuracy, PPV, NPV, and AUC of the combination of three assays with sequential mNGS (Dataset S4). Compared to the combination of Xpert, culture, and AFS (Xpert + culture + AFS), the addition of sequential mNGS (Xpert + culture + AFS + mNGS) significantly increased sensitivity from 51.2% to 70.4%, accuracy from 67.2% to 78.8%, and NPV from 51.3% to 63.3%, but specificity and PPV slightly decreased from 97.4% to 94.2% and from 97.4% to 95.7%, respectively (Fig. 4A and B). Adding mNGS increased sensitivity by 19.2 percentage points and decreased specificity by 3.2 percentage points; the number needed to diagnose (NND) (37, 38) to detect one additional true-positive case was 5.2. Noticeably, the AUCs of the three recommended assays with sequential mNGS were improved from 0.743 (95% CI, 0.776–0.868) to 0.823 (95% CI, 0.776–0.868). Thus, our study suggests that sequential mNGS was a powerful strategy for TB diagnosis (Fig. 4C).

To further evaluate the diagnostic performance of mNGS compared to Xpert combined with culture and AFS (referred to as conventional microbiological tests) across different clinical subgroups, a multilevel logistic regression analysis was conducted. Notably, tests for interaction across all subgroups were not statistically significant (all *P*interaction >0.05), indicating that the superior detection performance of mNGS over other conventional methods remained consistent among patients with different immune statuses, sexes, smoking histories, diabetic statuses, malignancies, and hypertension conditions, suggesting good generalizability (Fig. S3).

### Characterization of additional microorganisms detected by mNGS

**Fig 5:**
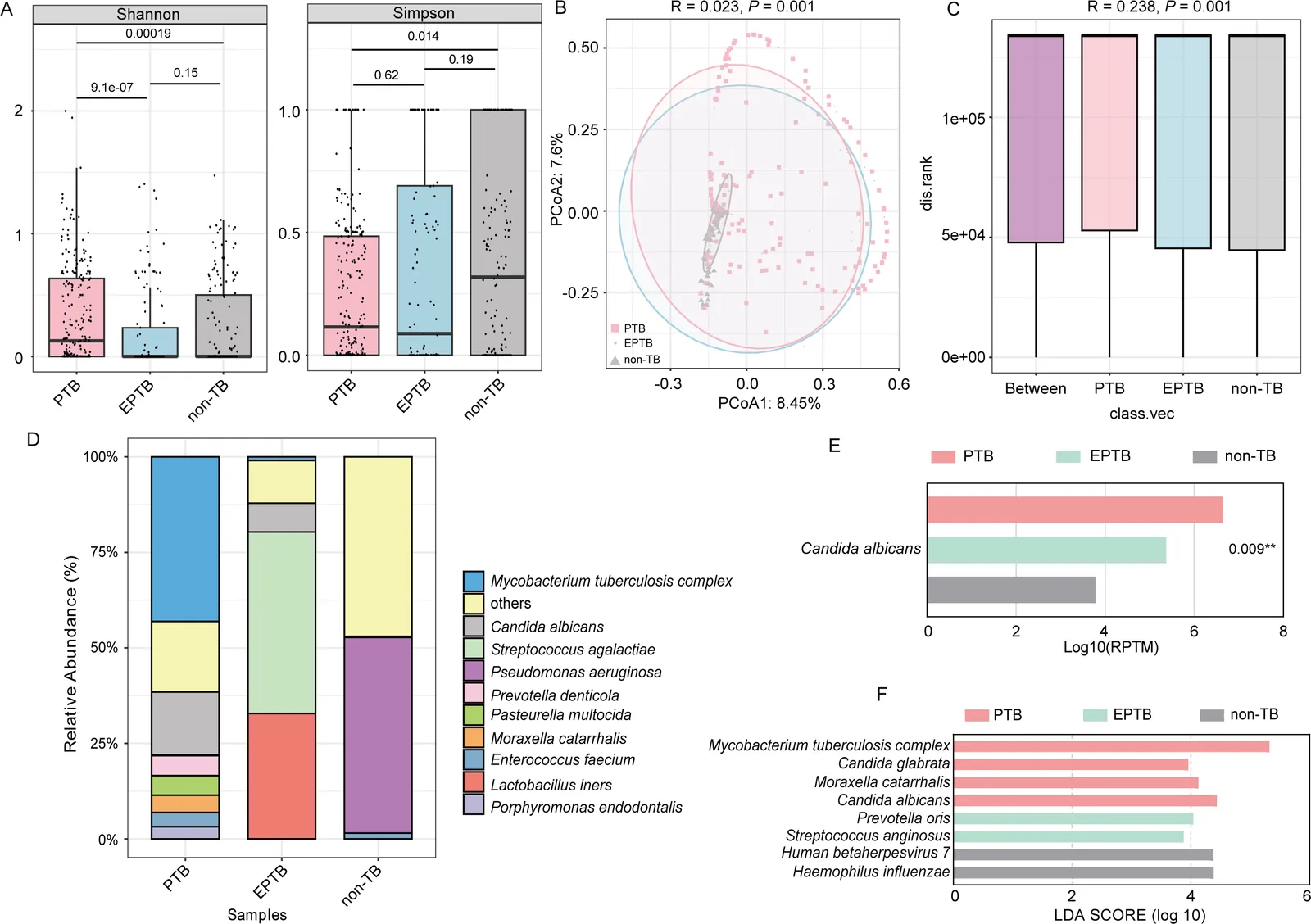
Diagnostic value of mNGS of the difference in microbial composition for patients with or without TB infection. (**A**) Alpha diversity is shown by Shannon and Simpson indices. (**B**) PCoA of the microbial composition. (**C**) ANOSIM for the analysis of microbial community structure. (**D**) Barplot shows the top 10 species with the highest abundance among three groups. (**E**) Analysis of significant differences of the species among the three groups with the Kruskal-Wallis test. (**F**) LEfSe analysis for enriched species in the three groups. **P < 0.01.

To characterize the additional microorganisms detected by mNGS alongside MTB, we analyzed a total of 558 mNGS results from the PTB (*n* = 133), EPTB (*n* = 250), and non-TB (*n* = 175) groups by using microbial community structure analysis (Dataset S5; Table 3). Significant differences between PTB and non-TB groups were observed according to both Shannon and Simpson indices, indicating differences in microbial richness and evenness (Fig. 5A). PCoA results showed that samples from both groups were intermixed; however, the infection group (PTB and EPTB) displayed a wider spread of data compared to the non-TB group (Fig. 5B). Moreover, a significant difference in microbial community structure among the three groups was observed (Fig. 5C).

**TABLE 3:** mNGS test characteristics associated with 3,757 samples from 3,645 patients tested from 2021 to 2024

Number (%) of mNGS detected organisms	Number (%) of patients (*n* = 3,757 samples)	Number (%) of PTB (*n* = 1,387 samples)	Number (%) of EPTB (*n* = 900 samples)	Number (%) of non-TB (*n* = 1,470 samples)
TB	210 (18.8)	164 (26)	46 (25.6)	0 (0)
Bacteria	276 (24.7)	138 (21.9)	38 (21.1)	100 (32.8)
Virus	478 (42.8)	233 (36.9)	83 (46.1)	162 (53.1)
Fungi	148 (13.3)	96 (15.2)	11 (6.1)	41 (13.4)
Parasite	4 (0.4)	0 (0)	2 (1.1)	2 (0.7)
All organisms	1,116 (100)	631 (100)	180 (100)	305 (100)

The relative abundances of the top 10 species were *Mycobacterium tuberculosis* complex (total reads = 11,553,832), *Candida albicans* (total reads = 4,830,142), *Streptococcus agalactiae* (total reads = 1,496,955), *Pseudomonas aeruginosa* (total reads = 1,478,854), *Prevotella denticola* (total reads = 1,404,349), *Pasteurella multocida* (total reads = 1,373,420), *Moraxella catarrhalis* (total reads = 1,207,763), *Enterococcus faecium* (total reads = 1,038,650), *Lactobacillus iners* (total reads = 1,033,137), and *Porphyromonas endodontalis* (total reads = 855,292). Several of these are common respiratory commensals. Among them, only *Candida albicans* showed a significantly different amount of mNGS reads among the three groups (Fig. 5D and E). Additionally, LEfSe analysis revealed that there were significantly more abundant *Candida albicans*, *Moraxella catarrhalis*, *Candida glabrata*, and *Mycobacterium tuberculosis* complex in the PTB group, *Streptococcus anginosus* and *Prevotella oris* in the EPTB group, and *Haemophilus influenzae* and Human betaherpesvirus 7 in the non-TB group (Fig. 5F).

### Cost-effectiveness analyses of mNGS for TB diagnosis

**Fig 6:**
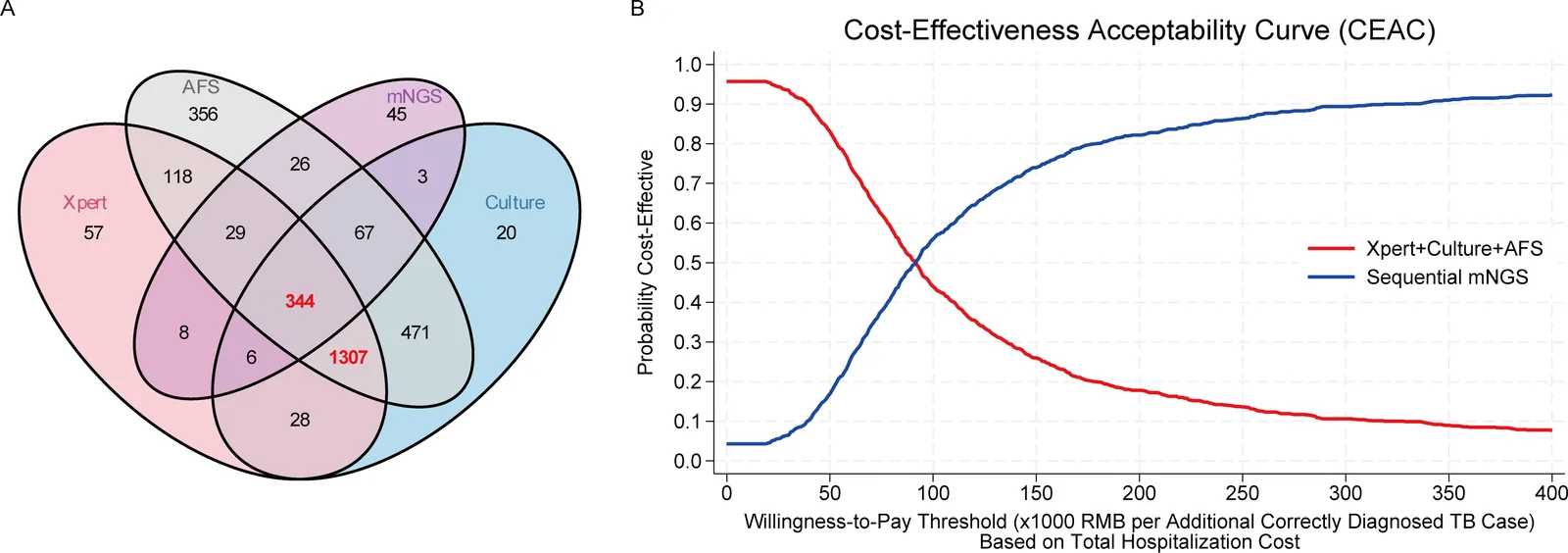
Cost-effectiveness acceptability curve for the detection methods. (**A**) The number of methods done in clinic by one, two, three, and all four methods, including mNGS, Xpert, culture, and AFS, in 3,757 patients are shown in the Venn diagram. (**B**) Cost-effectiveness acceptability curves for the mNGS method of TB diagnosis.

In our study, 1,307 out of 3,757 participants received three WHO-recommended assays, AFS, culture, and Xpert, while only 344 out of 1,307 participants received a sequential mNGS (Fig. 6A). The total medical care cost of the WHO-recommended strategy was around 23,938 ± 47,328 RMB, while that of a sequential mNGS was 27,284 ± 26,650 RMB (Table 4). Consistent results showing the same trend were also observed in TB subtype PTB and EPTB (Table S2). Thus, it indicated that the strategy with a sequential mNGS should apply to patients with higher medical costs, although a sequential mNGS provided advantages in diagnostic performance for TB and identification of untargeted co-infecting pathogens over the WHO-recommended assays.

**TABLE 4:** Total cost analyses of Xpert + culture + AFS and Xpert + culture + AFS + mNGS for TB infection^*b*^

Test	*N*	Mean (SD), ¥	Median (IQR), ¥	Adjusted mean^*a*^ (95%CI), ¥	Adjusted mean difference (95%CI) (mNGS vs Other 3)	Adjusted relative ratio
Xpert + culture + AFS	1,307	23,938 (47,328)	16,005 (11,967, 22,123)	23,293 (21,490, 25,096)	4,394 (1,927, 6,861), *P* < 0.001	1.19 (1.08, 1.31), *P* < 0.001
Xpert + culture + AFS + mNGS	344	27,284 (26,650)	19,540 (16,305, 27,293)	27,687 (25,155, 30,220)		

^*a*^ We used a GLM with Gamma family and log link, adjusting for demographic (age, sex, and smoking), comorbidities (diabetes, tumor, hypertension, and immunosuppression), surgery status, and inflammatory marker categories. Cluster-robust standard errors were used at the inpatient ID level.

^*b*^ SD, standard deviation; IQR, interquartile range (25th percentile, 75th percentile); and CI, confidence interval.

Then, to determine whether clinical application of a sequential mNGS could improve medical care efficiency for TB diagnosis, we conducted a cost-utility analysis of the two strategies. The CEAC illustrated the cost-effectiveness of the WHO-recommended assays with (blue curve) or without (red curve) a sequential mNGS at various willingness-to-pay thresholds (Fig. 6B). At lower WTP thresholds (below 50,000 RMB per additional correctly diagnosed TB case), the WHO-recommended strategy was 95% cost-effective compared to the strategy with a sequential mNGS. However, a sequential mNGS becomes the optimal cost-effective choice with near-certain probability (>80%) at a WTP threshold approximately 200,000 RMB per additional correctly diagnosed TB case. Sequential mNGS becomes the optimal cost-effective choice, with near-certain probabilities of >90% and >70% at WTP thresholds of approximately 150,000 RMB (for PTB) and 300,000 RMB (for EPTB) per additional correctly diagnosed case, respectively (Fig. S4 and S5).

## DISCUSSION

In this study, we observed significant differences in gender, diabetes, tumors, hypertension, PCT, hsCRP, immunosuppression, lymphocyte counts, lymphocyte percentage, IFN-γ, ESR, CD4+ T cells, and CD8+ T cells among the PTB, EPTB, and non-TB groups (*P* < 0.05) (Table 1), which were consistent with our Spearman correlation analyses and the previous reports by WHO (2) and other groups (39–41). The top four frequently requested assays were AFS, culture, TB-IGRA, and Xpert, which demonstrated that physicians in our hospital routinely relied on the WHO-recommended methods, including AFS, culture, and Xpert for MTB detection (2). mNGS was most rarely requested for highly suspicious cases with negative results in other TB assays (42–44). Noticeably, the EPTB group with 24% participation received nearly half of the total TB assay requests, suggesting that the diverse clinical manifestations of EPTB, the difficulty in obtaining pathogenic specimens, and the overall complexity of its diagnosis may drive clinicians to more frequently request multiple assays to improve diagnostic accuracy.

Additionally, our study demonstrated that mNGS possessed superior performance for TB diagnosis among seven TB diagnostic assays, such as the highest specificity (100%), accuracy (72.3%), and AUC value (0.795) but lower sensitivity (59.0%) than that of TB-IGRA (79.5%). However, TB-IGRA, with its low specificity (17.8%) and PPV (54.3%), had a high risk of false positives in specific clinical contexts, limiting its standalone diagnostic utility. Furthermore, subgroup analyses showed that the diagnostic performance of mNGS was comparable to that of conventional methods across patients with varying immune statuses, sexes, smoking histories, diabetic statuses, malignancies, and hypertension, indicating consistent generalizability. Further analysis of mNGS performance across different specimen types revealed its heterogeneity in diagnostic abilities. Both specificity and PPV were 100% for all specimen types. Its sensitivity was significantly higher in BALF (71.0%) and tissue (72.7%) compared to CSF (36.5%) and other specimen types. The accuracy (81.6%), NPV (66.7%), and AUC (0.855; 95% CI, 0.816–0.894) in BALF specimens were much higher than those in other specimen types. Thus, it suggests that BALF was an optimal specimen type for mNGS in the diagnosis of TB, particularly PTB (45, 46). For non-BALF specimen types, further prospective studies with expanded specimen sizes are needed to achieve more accurate estimates of diagnostic accuracy (Table S3).

AFS, culture, and Xpert have been recommended as routine assays for TB diagnosis by WHO. In our study, we demonstrated that a sequential mNGS could significantly increase its diagnostic sensitivity from 51.2% to 70.4% (47), accuracy from 67.2% to 78.8%, NPV from 51.3% to 63.3%, and AUC from 0.743 to 0.823. The incremental diagnostic benefit of adding mNGS was driven primarily by a substantial gain in sensitivity (+19.2 percentage points), with only a modest decrease in specificity (–3.2 percentage points). This sensitivity improvement is clinically meaningful, as it allows the detection of one additional true-positive case for every five to six patients tested (NND = 5.2). In the context of TB control, reducing false negatives outweighs the small increase in false positives, especially when positive mNGS results can be corroborated by clinical and imaging findings. Therefore, a sequential mNGS testing strategy is a powerful approach to improve TB diagnosis, particularly for challenging cases with negative results from other TB assays due to low bacterial load or atypical presentation.

The superior sensitivity of mNGS in this study stems from its core methodologies. Its mechanical bead-beating step facilitates more efficient DNA release from tough-walled mycobacteria than chemical lysis alone (48). Furthermore, deep, untargeted sequencing enables detection of low-abundance pathogens without relying on single-target amplification, conferring an advantage in paucibacillary samples like smear-negative or extrapulmonary TB (49). Additionally, our stringent contamination control protocols (physical separation, batch controls, background read removal, and retesting upon suspicion) effectively minimized false positives, strengthening confidence in the mNGS results presented. In contrast, Xpert MTB/RIF, while excellent for rapid, targeted detection, approaches its sensitivity limit in low-bacterial-load contexts, a recognized constraint per WHO guidelines (18, 50). We acknowledge the trade-offs: mNGS’s sensitivity comes with higher cost, complexity, and contamination risk, whereas Xpert’s speed, standardization, and direct resistance information remain vital for frontline use. Thus, our findings do not supplant Xpert but highlight mNGS’s complementary value in complex cases. The performance difference reflects their distinct design paradigms: “precision targeting” versus “broad surveillance.”

Our analysis also revealed a significant disruption of the respiratory microbiota in TB patients by mNGS. The reduced alpha diversity in the PTB group indicated a loss of microbial richness and evenness in association with MTB infection. Although the microbial communities were partially intermixed, the greater dispersion in the TB groups suggests increased compositional heterogeneity. The relative abundance of the most common species was similar across groups, with *Candida albicans* being a notable differentially abundant species. Some studies have reported a higher prevalence of *Candida albicans* detection in respiratory samples from TB patients (51, 52). However, differentiating between colonization, contamination, and true co-infection remains challenging. Therefore, whether TB infection truly facilitates opportunistic co-infections or merely alters the microbial landscape without pathogenic consequences is currently unclear. The clinical significance of these microbial shifts requires further investigation.

In previous studies, the clinical utility and cost-effectiveness of mNGS were recommended to elucidate its precise clinical indications and determine the optimal timing for its application (53). The extant literature has predominantly concentrated on comparing the charging standards of detection methods themselves (54). This study, however, constitutes the first investigation to assess aggregate inpatient expenditure and diagnostic fees in cohorts who received sequential mNGS versus those who did not (55, 56). For the first time, the cost-effectiveness analysis demonstrated a clear economic advantage of sequential mNGS over without mNGS for TB across a wide range of willingness-to-pay thresholds. At lower WTP thresholds (50,000 RMB), the WHO-recommended assays demonstrated superior cost-effectiveness, supporting their widespread implementation in resource-limited settings. When WTP exceeds approximately 200,000 RMB per case, sequential mNGS becomes more cost-effective. Sequential mNGS is more cost-effective at higher WTP thresholds, with a lower threshold and a more pronounced advantage for PTB. This phenomenon may be attributed to the fact that more complex conditions are associated with a higher number of complications and greater medical costs, especially for EPTB. While this reflects the increased resource utilization in severe cases, it also underscores the diagnostic value of NGS for complex diseases and extrapulmonary tuberculosis. This transition supports employing mNGS for diagnostically challenging cases or in well-resourced institutions, where its enhanced diagnostic capability offers economic advantages through improved diagnostic accuracy and treatment optimization. These findings advocate for a tiered diagnostic approach: prioritizing the WHO-recommended assays in basic healthcare settings while reserving sequential mNGS for complex cases or advanced medical institutions, thus optimizing resource allocation in tuberculosis control.

Even with meticulous design and analysis, our study also had some limitations. First, not all patients underwent seven TB assays because of the nature of retrospective clinical studies, which led to a lack of corresponding comparative diagnostic performance results. Second, our study was a single-center study with a relatively limited sample size in particular specimen types, which may cause bias in the analysis outcomes. Third, the sample sizes for non-BALF specimen types (e.g., CSF, blood, tissue, and body fluid) were relatively small, leading to imprecise sensitivity estimates. Therefore, these results are hypothesis-generating and require validation in larger prospective studies (Table S3). Fourth, because our proposed tiered diagnostic approach was evaluated in a single tertiary referral center, its generalizability to other healthcare settings (e.g., primary care facilities, district hospitals, or regions with different TB epidemiology) remains uncertain. External validation in diverse clinical and geographic settings is needed to confirm the robustness and reproducibility of our conclusions before widespread implementation.

### Conclusion

In summary, this large-scale retrospective study demonstrates that mNGS is a highly specific and clinically reliable diagnostic tool for detecting *Mycobacterium tuberculosis* across diverse specimen types and patient age groups. With its superior sensitivity, particularly in extrapulmonary tuberculosis, and perfect specificity, mNGS offers considerable diagnostic value, especially in complex or culture-negative cases. Furthermore, a sequential mNGS after conventional TB assays such as AFS, culture, and Xpert significantly enhances diagnostic sensitivity and overall accuracy. Microbial profiling also revealed significant intergroup differences in organisms such as *Candida albicans*, suggesting potential clinical relevance. From a health-economic perspective, sequential mNGS proves increasingly cost-effective with higher willingness-to-pay thresholds. These findings support the integration of mNGS into routine TB diagnostic algorithms to complement existing assays and improve early, accurate diagnosis.

## Supplementary Material

Reviewer comments

## Data Availability

The data that support the findings of this study are available from the corresponding author upon reasonable request. No new nucleotide or amino acid sequences were generated in this retrospective study.

## SUPPLEMENTAL MATERIAL

Data set S1 spectrum.00970-26-s0001.xlsx Detailed information of Figure 1.

Data set S2 spectrum.00970-26-s0002.xlsx Detailed information of Figure 2.

Data set S3 spectrum.00970-26-s0003.xlsx Detailed information of Figure 3.

Data set S4 spectrum.00970-26-s0004.xlsx Detailed information of Figure 4.

Data set S5 spectrum.00970-26-s0005.xlsx Detailed information of Figure 5.

Supplemental figures and tables spectrum.00970-26-s0006.docx Fig. S1 to S5 and Tables S1 to S3.

OPEN PEER REVIEW reviewer-comments.pdf An accounting of the reviewer comments and feedback.

ASM does not own the copyrights to Supplemental Material that may be linked to, or accessed through, an article. The authors have granted ASM a non-exclusive, world-wide license to publish the Supplemental Material files. Please contact the corresponding author directly for reuse.
