# Supplementary material for: Metagenomic next-generation sequencing for tuberculosis diagnosis: enhanced performance and cost-effectiveness
Source: Microbiol Spectr. 2026 Jul 29;14(9):e00970-26. doi: 10.1128/spectrum.00970-26 (PMC13532181; doi:10.1128/spectrum.00970-26)
Supplement: Supplementary file 1 [file reviewer-comments.pdf]

AMERICAN SOCIETY FOR MICROBIOLOGY | Microbiology Spectrum

Metagenomic Next-Generation Sequencing for Tuberculosis Diagnosis: Enhanced Performance and Cost-Effectiveness

Xiaoli Zhou, Guo Wei, Tiange Song, Yanqiu Yu, Jianwei Chen, Juan Long, Xiaoyan Tao, Jie Zhang, and Li Jiang

Corresponding Author(s): Li Jiang, Sichuan Academy of Medical Sciences & Sichuan Provincial People's Hospital

Review Timeline:

Submission Date:	March 19, 2026
Editorial Decision:	May 31, 2026
Revision Received:	June 20, 2026
Accepted:	June 22, 2026

Editor: Benjamin Liu

Reviewer(s): Disclosure of reviewer identity is with reference to reviewer comments included in decision letter(s). The following individuals involved in review of your submission have agreed to reveal their identity: Ranu Soni (Reviewer #3)

Transaction Report:

(Note: With the exception of the correction of typographical or spelling errors that could be a source of ambiguity, letters and reports are not edited. The original formatting of letters and referee reports may not be reflected in this compilation.)

DOI: https://doi.org/10.1128/spectrum.00970-26

Re: Spectrum00970-26 (**Metagenomic Next-Generation Sequencing for Tuberculosis Diagnosis: Enhanced Performance and Cost-Effectiveness**)

Dear Dr. Li Jiang:

Thank you for the privilege of reviewing your work. Below you will find my comments, instructions from the Spectrum editorial office, and the reviewer comments.

Editor's comments:

The authors are suggested to introduce that a nucleic acid amplification test, that is a tuberculosis (TB)-LAMP-based assay, was added to the first World Health Organization (WHO) List of Essential In Vitro Diagnostics, and recommended for the diagnosis of active pulmonary tuberculosis in the primary care setting. More references should be cited, with this one (Liu, B.M. Isothermal nucleic acid amplification technologies and CRISPR-Cas based nucleic acid detection strategies for infectious disease diagnostics. p 30-47. In Manual of Molecular Microbiology: Fundamentals and Applications; ASM Press: Washington, DC, USA, 2025.doi:10.1128/9781683674597.ch03.) as an example (citing is optional).

Please return the manuscript within 30 days; if you cannot complete the modification within this time period, please contact me. If you do not wish to modify the manuscript and prefer to submit it to another journal, notify me immediately so that the manuscript may be formally withdrawn from consideration by Spectrum.

Revision Guidelines
To submit your modified manuscript, log into the submission site at https://spectrum.msubmit.net/cgi-bin/main.plex. Go to Author Tasks and click the appropriate manuscript title to begin. The information you entered when you first submitted the paper will be displayed; update this as necessary. Note the following requirements:

• Upload point-by-point responses to the issues raised by the reviewers in a file named "Response to Reviewers," NOT in your cover letter.
• Upload a compare copy of the manuscript (without figures) as a "Marked-Up Manuscript" file.
• Upload a clean .DOC/.DOCX version of the revised manuscript and remove the previous version.
• Each figure must be uploaded as a separate, editable, high-resolution file (TIFF or EPS preferred), and any multipanel figures must be assembled into one file.
• Any supplemental material intended for posting by ASM should be uploaded with their legends separate from the main manuscript. You can combine all supplemental material into one file (preferred) or split it into a maximum of 10 files with all associated legends included.

For complete guidelines on revision requirements, see our Submission and Review Process webpage. Submission of a paper that does not conform to guidelines may delay acceptance of your manuscript.

Data availability: ASM policy requires that data be available to the public upon online posting of the article, so please verify all links to sequence records, if present, and make sure that each number retrieves the full record of the data. If a new accession number is not linked or a link is broken, provide Spectrum production staff with the correct URL for the record. If the accession numbers for new data are not publicly accessible before the expected online posting of the article, publication may be delayed; please contact production staff (Spectrum@asmusa.org) immediately with the expected release date.

Publication Fees: For information on publication fees and which article types are subject to charges, visit our website. If your manuscript is accepted for publication and any fees apply, you will be contacted separately about payment during the production process; please follow the instructions in that e-mail. Arrangements for payment must be made before your article is published.

ASM Membership: Corresponding authors may join or renew ASM membership to obtain discounts on publication fees. Need to upgrade your membership level? Please contact Customer Service at Service@asmusa.org.

The ASM Journals program strives for constant improvement in our submission and publication process. Please tell us how we can improve your experience by taking this quick Author Survey.

Thank you for submitting your paper to Spectrum.

Sincerely,
Benjamin Liu
Editor

Reviewer #1 (Comments for the Author):

This study evaluates seven tuberculosis diagnostic assays including metagenomic next-generation sequencing (mNGS), tuberculosis IgG antibody (TB-IgG), tuberculosis interferon-γ release assay (TB-IGRA), TB DNA, Xpert MTB/RIF, culture, and acid-fast bacilli staining (AFS) in a large retrospective cohort of clinically suspected TB patients. The authors report that mNGS demonstrated the highest specificity, accuracy, and AUC among the tested assays, and that sequential use of mNGS improved overall diagnostic performance and became cost-effective at higher willingness-to-pay thresholds. The manuscript addresses an important and clinically relevant topic, particularly the role of mNGS in diagnostically challenging tuberculosis cases. Inclusion of multiple conventional and molecular assays in a large retrospective cohort is a strength.
However, some issues should be addressed before publication.
1. The association of Candida albicans with TB co-infection should be interpreted cautiously, as Candida species are commonly detected as commensal colonizers in respiratory samples, and differentiation between commensal colonization, contamination, and true infection remains difficult. Similarly, Figure 5D and 5F should be interpreted cautiously, as several detected organisms are commonly associated with commensal microbiota rather than confirmed pathogenic co-infection. The clinical significance of the additional microorganisms detected by mNGS remains unclear. It would be helpful to clarify whether these findings influenced clinical management or antimicrobial/antifungal treatment decisions in these patients.
2. WHO has previously discouraged the use of commercial serological antibody tests, including TB-IgG assays, for diagnosis of active tuberculosis because of inconsistent sensitivity and specificity and potential false-positive results. The rationale for including TB-IgG in the diagnostic comparison should therefore be better justified.
3. In Figure 4A, the combination of Xpert MTB/RIF + culture + AFS already demonstrates very high specificity (97.4%). The incremental benefit of adding mNGS appears mainly sensitivity-related and should be quantitatively discussed.
4. The relatively high prevalence of tumors among the study patients, particularly in a cohort of suspected TB cases, warrants further explanation, as malignancy-associated immunosuppression can strongly influence immune parameters, IGRA performance, microbial colonization, opportunistic infections findings, thereby potentially affecting diagnostic interpretation.
4. Minor language revisions are recommended throughout the manuscript to improve grammar, readability, and consistency of terminology. Examples include:
(i) Line 62: "lading" should be corrected to "leading"
(ii) Line 131: The term "wall-breaking procedure" is non-standard and should be replaced with a more appropriate scientific term
(iii) Lines 145 and 149: "mNSG" should be corrected to "mNGS"
(iv) Line 264 and Figure 5D: "Morasella catarrhalis" should be corrected to Moraxella catarrhalis
(v) Figure 5D and 5E: Species names should be italicized according to scientific nomenclature conventions
(vi) Line 314: "cultur" should be corrected to "culture"

Reviewer #3 (Comments for the Author):

• Clarify reference standard construction: Provide explicit details on how the composite reference standard was constructed, including adjudication procedures for discordant results.
• Statistical power analysis: Include a priori sample size calculations to justify the adequacy of specimen-type-specific cohorts.
• Supplementary data: Consider providing detailed supplementary tables with specimen-type-specific performance metrics and stratified cost-effectiveness analyses by TB subtype (PTB vs. EPTB).
• Discussion of contamination: Expand the discussion with specific mNGS contamination rates observed in this cohort and describe decontamination protocols employed.
• External validation: Briefly discuss the need for external validation in diverse healthcare settings to confirm the proposed tiered diagnostic approach.

This study evaluates seven tuberculosis diagnostic assays including metagenomic next-generation sequencing (mNGS), tuberculosis IgG antibody (TB-IgG), tuberculosis interferon-γ release assay (TB-IGRA), TB DNA, Xpert MTB/RIF, culture, and acid-fast bacilli staining (AFS) in a large retrospective cohort of clinically suspected TB patients. The authors report that mNGS demonstrated the highest specificity, accuracy, and AUC among the tested assays, and that sequential use of mNGS improved overall diagnostic performance and became cost-effective at higher willingness-to-pay thresholds. The manuscript addresses an important and clinically relevant topic, particularly the role of mNGS in diagnostically challenging tuberculosis cases. Inclusion of multiple conventional and molecular assays in a large retrospective cohort is a strength.

However, some issues should be addressed before publication.

1. The association of Candida albicans with TB co-infection should be interpreted cautiously, as Candida species are commonly detected as commensal colonizers in respiratory samples, and differentiation between commensal colonization, contamination, and true infection remains difficult. Similarly, Figure 5D and 5F should be interpreted cautiously, as several detected organisms are commonly associated with commensal microbiota rather than confirmed pathogenic co-infection. The clinical significance of the additional microorganisms detected by mNGS remains unclear. It would be helpful to clarify whether these findings influenced clinical management or antimicrobial/antifungal treatment decisions in these patients.

2. WHO has previously discouraged the use of commercial serological antibody tests, including TB-IgG assays, for diagnosis of active tuberculosis because of inconsistent sensitivity and specificity and potential false-positive results. The rationale for including TB-IgG in the diagnostic comparison should therefore be better justified.

3. In Figure 4A, the combination of Xpert MTB/RIF + culture + AFS already demonstrates very high specificity (97.4%). The incremental benefit of adding mNGS appears mainly sensitivity-related and should be quantitatively discussed.

4. The relatively high prevalence of tumors among the study patients, particularly in a cohort of suspected TB cases, warrants further explanation, as malignancy-associated

immunosuppression can strongly influence immune parameters, IGRA performance, microbial colonization, opportunistic infections findings, thereby potentially affecting diagnostic interpretation.

4. Minor language revisions are recommended throughout the manuscript to improve grammar, readability, and consistency of terminology. Examples include:

(i) Line 62: “lading” should be corrected to “leading”

(ii) Line 131: The term “wall-breaking procedure” is non-standard and should be replaced with a more appropriate scientific term

(iii) Lines 145 and 149: “mNSG” should be corrected to “mNGS”

(iv) Line 264 and Figure 5D: “Morasella catarrhalis” should be corrected to *Moraxella catarrhalis*

(v) Figure 5D and 5E: Species names should be italicized according to scientific nomenclature conventions

(vi) Line 314: “cultur” should be corrected to “culture”

PEER REVIEW REPORT

Journal: Microbiology Spectrum (American Society for Microbiology)

Manuscript Title: Metagenomic Next-Generation Sequencing for Tuberculosis Diagnosis: Enhanced Performance and Cost-Effectiveness

Review Date: 05/04/2026

1. SUMMARY OF THE STUDY

This retrospective observational study evaluates the diagnostic performance and cost-effectiveness of metagenomic next-generation sequencing (mNGS) in the diagnosis of tuberculosis (TB). 1 The authors analyzed 16,776 results from seven TB diagnostic assays (mNGS, TB-IgG, TB-IGRA, TB-DNA, Xpert MTB/RIF, Culture, and acid-fast bacilli staining) in 3,757 patients suspected of TB infection at Sichuan Provincial People's Hospital from September 2021 to July 2024. 2 The study demonstrates that mNGS achieves superior specificity (100%), accuracy (72.3%), and AUC (0.795), with sensitivity of71.0% in BALF and 72.7% in tissue samples. 1 When used sequentially after conventional WHO-recommended tests, mNGS improved diagnostic sensitivity to 70.4% and AUC to 0.823. 34 A cost-effectiveness analysis revealed that sequential mNGS becomes cost-effective at willingness-to-pay thresholds exceeding 200,000 RMB per correct diagnosis. 1

2. STRENGTHS

2.1 Comprehensive Study Design

- **Large sample size**: The study includes 16,776 assay results from 3,757 participants, providing robust statistical power for meaningful comparisons across multiple diagnostic modalities. 1
- **Systematic comparison**: The simultaneous evaluation of seven TB diagnostic assays offers a thorough comparative framework, establishing mNGS's relative performance in clinical practice. 1
- **Multiple specimen types**: The analysis across BALF, tissue, CSF, sputum, and other sample types provides clinically relevant insights into assay performance variability. 53

2.2 Novel Economic Analysis

- **First comprehensive cost-effectiveness study:** The authors acknowledge this is the first investigation to assess aggregate inpatient expenditure alongside diagnostic

performance, rather than merely comparing assay costs. 67 This addresses a significant gap in the literature and provides critical health-economic evidence for clinical implementation.

- **Tiered diagnostic approach recommendation:** The proposed strategy—prioritizing WHO-recommended assays in resource-limited settings while reserving sequential mNGS for well-resourced institutions—is pragmatic and implementable. 8

2.3 Mechanistic Insights

- **Microbiological profiling**: Beyond diagnostic performance, the study reveals disruptions in respiratory microbiota in TB patients, identifying Candida albicans as a key co-infection marker. 96 This finding enhances understanding of TB disease pathogenesis and potential biomarkers.
- **Technical explanation:** The discussion thoroughly explains why mNGS outperforms Xpert MTB/RIF in low-bacterial-load contexts, highlighting the efficiency of bead-beating DNA extraction and untargeted sequencing advantages. 4

2.4 Ethical Compliance

The study received appropriate ethics approval with clear disclosure of a waiver for informed consent due to the retrospective nature. 10,11

3. LIMITATIONS

3.1 Study Design Limitations

- **Incomplete assay coverage:** Not all patients underwent all seven TB assays, limiting the ability to conduct comprehensive comparative diagnostic performance analyses. 810 This is a significant limitation that undermines the comprehensiveness of inter-assay comparisons and introduces verification bias.
- **Single-center retrospective design**: The single-center nature of a specialized hospital in Sichuan Province may limit generalizability to other geographic regions, healthcare settings, and diverse patient populations. 10 The retrospective design introduces inherent confounders and selection bias that cannot be fully controlled.
- **Limited specimen type sizes:** The authors acknowledge that particular specimen types had limited sample sizes, which may bias analyses, especially for CSF, where sensitivity was notably lower (36.5%). 310

3.2 Methodological Considerations

- **Composite reference standard**: The study does not explicitly detail the construction and validation of the composite reference standard used to evaluate diagnostic performance. Clarity on how discordant results were adjudicated would strengthen the analysis.
- **Cost-effectiveness threshold variability:** The cost-effectiveness threshold (200,000 RMB per correct diagnosis) may not be universally applicable across different healthcare systems and economic contexts, limiting transferability.

3.3 Clinical Applicability Gaps

- **Turnaround time not addressed**: While cost-effectiveness is discussed, the turnaround time for mNGS results compared to conventional assays is not explicitly addressed, which is critical for clinical decision-making in TB diagnosis.
- **Contamination risk acknowledged but not quantified**: The authors acknowledge higher contamination risk with mNGS but do not provide specific contamination rates or mitigation strategies. 49

4. MAJOR ISSUES AND SUGGESTIONS FOR REVISION

- **Clarify reference standard construction**: Provide explicit details on how the composite reference standard was constructed, including adjudication procedures for discordant results.
- **Statistical power analysis**: Include a priori sample size calculations to justify the adequacy of specimen-type-specific cohorts.
- **Supplementary data**: Consider providing detailed supplementary tables with specimen-type-specific performance metrics and stratified cost-effectiveness analyses by TB subtype (PTB vs. EPTB).
- **Discussion of contamination**: Expand the discussion with specific mNGS contamination rates observed in this cohort and describe decontamination protocols employed.
- **External validation**: Briefly discuss the need for external validation in diverse healthcare settings to confirm the proposed tiered diagnostic approach.

5. ASSESSMENT AND RECOMMENDATION

Strengths outweigh limitations. This is a rigorous, large-scale study addressing an important clinical question with novel economic insights. The demonstration that sequential mNGS significantly enhances diagnostic sensitivity (70.4% vs. 51.2% with WHO-recommended assays alone) 3 provides compelling evidence for its integration into diagnostic algorithms, particularly for extrapulmonary and culture-negative TB cases. 10 The cost-effectiveness analysis at higher willingness-to-pay thresholds offers valuable guidance for resource allocation in TB control programs.

The paper makes meaningful contributions to the field by:

- Establishing mNGS's superior specificity and comparable sensitivity for TB diagnosis across multiple specimen types
- Providing the first comprehensive health-economic analysis integrating diagnostic performance with actual healthcare costs
- Proposing an implementable tiered diagnostic strategy balancing resource constraints and clinical utility

Recommendation: ACCEPT WITH MAJOR REVISIONS

This manuscript is suitable for publication in Microbiology Spectrum. It addresses an important clinical diagnostic challenge with robust evidence, novel economic analysis, and clear practical implications. The limitations are acknowledged transparently and do not significantly undermine the conclusions. After addressing the minor revisions suggested above, the manuscript will be a valuable contribution to TB diagnostic literature.

Dear Editor and Reviewers,

We are very grateful that the editors and reviewers gave us valuable comments and suggestions. We have revised the manuscript carefully under the guidance of your excellent reviewers. All changes made in the revised manuscript have been highlighted in the text. Hereby, we enclosed point-to-point responses to the comments raised by the reviewers as followings.

We believe that the revisions made in response to the comments from the editor and reviewers have improved the manuscript, and hope that the revised manuscript will be found to be suitable for publication.

Sincerely,

Li Jiang for all the coauthors

Response to Editor

Major comments:

The authors are suggested to introduce that a nucleic acid amplification test, that is a tuberculosis (TB)-LAMP-based assay, was added to the first World Health Organization (WHO) List of Essential In Vitro Diagnostics, and recommended for the diagnosis of active pulmonary tuberculosis in the primary care setting. More references should be cited, with this one (Liu, B.M. Isothermal nucleic acid amplification technologies and CRISPR-Cas based nucleic acid detection strategies for infectious disease diagnostics. p 30-47. In Manual of Molecular Microbiology: Fundamentals and Applications; ASM Press: Washington, DC, USA, 2025.doi:10.1128/9781683674597.ch03.) as an example (citing is optional).

Response:

Yes, we greatly appreciate the important comment raised by editor. As recommended, we have added a statement at lines 79–83 in the Introduction and cited the 2018 WHO Essential Diagnostics List and reference.

"Additionally, the 2018 WHO Essential Diagnostics List included TB-LAMP for pulmonary tuberculosis in primary care. Its advantages include simplicity, rapid results, and the ability to replace sputum smear microscopy as an initial test. However, the recommendation is conditional and based on very low-quality evidence, highlighting the need for further validation [20, 21]. "

Response to Reviewer #1

Major comments:

This study evaluates seven tuberculosis diagnostic assays including metagenomic next-generation sequencing (mNGS), tuberculosis IgG antibody (TB-IgG), tuberculosis interferon-γ release assay (TB-IGRA), TB DNA, Xpert MTB/RIF, culture, and acid-fast bacilli staining (AFS) in a large retrospective cohort of clinically suspected TB patients. The authors report that mNGS demonstrated the highest specificity, accuracy, and AUC among the tested assays, and that

sequential use of mNGS improved overall diagnostic performance and became cost-effective at higher willingness-to-pay thresholds. The manuscript addresses an important and clinically relevant topic, particularly the role of mNGS in diagnostically challenging tuberculosis cases. Inclusion of multiple conventional and molecular assays in a large retrospective cohort is a strength. However, some issues should be addressed before publication.

Response:

Thank you very much for your recognition of our research work.

Following are the major concerns of this study:

1. The association of Candida albicans with TB co-infection should be interpreted cautiously, as Candida species are commonly detected as commensal colonizers in respiratory samples, and differentiation between commensal colonization, contamination, and true infection remains difficult. Similarly, Figure 5D and 5F should be interpreted cautiously, as several detected organisms are commonly associated with commensal microbiota rather than confirmed pathogenic co-infection. The clinical significance of the additional microorganisms detected by mNGS remains unclear. It would be helpful to clarify whether these findings influenced clinical management or antimicrobial/antifungal treatment decisions in these patients.

Response:

Yes, we thank the reviewer for this important and constructive comment. We agree that the detection of Candida albicans and other commensal microorganisms in respiratory specimens should be interpreted with caution, as differentiation between colonization, contamination, and true infection is indeed challenging. Indeed, upon reviewing cases in which other microorganisms were detected, we found that no antibacterial or antifungal treatment was administered clinically, because once tuberculosis infection was confirmed, patients were immediately transferred to an infectious disease hospital. Therefore, to address this concern, we have made the following revisions:

1) Revised the following sentence at lines 42 in the Abstract section:

"Microbial analysis associated *Candida albicans* with TB ~~co-infection~~."

2) **Revised the following paragraphs at lines 281–302 in the Result section:**

"3. Characterization of additional microorganisms detected by mNGS

To characterize the additional microorganisms detected by mNGS alongside MTB, we analyzed a total of 558 mNGS results from the PTB (n=133), EPTB (n=250) and non-TB (n=175) groups by using microbial community structure analysis (Dataset S5, table 3). Significant differences between PTB and non-TB groups were observed according to both Shannon and Simpson indices, indicating differences in microbial richness and evenness (Fig. 5A). PCoA results showed that samples from both groups were intermixed; however, the infection group (PTB and EPTB) displayed a wider spread of data compared to the non-TB group (Fig. 5B). Moreover, a significant difference in microbial community structure among the three groups was observed (Fig. 5C). The relative abundances of the top 10 species were *Mycobacterium tuberculosis complex* (Total reads=11553832), *Candida albicans* (Total reads=4830142), *Streptococcus agalactiae* (Total reads=1496955), *Pseudomonas aeruginosa* (Total reads=1478854), *Prevotella denticola* (Total reads=1404349), *Pasteurella multocida* (Total reads=1373420), *Moraxella catarrhalis* (Total reads=1207763), *Enterococcus faecium* (Total reads=1038650), *Lactobacillus iners* (Total

reads=1033137), and *Porphyromonas endodontalis* (Total reads=855292). Several of these are common respiratory commensals. Among them, only *Candida albicans* showed significantly different amount of mNGS reads among the three groups (Fig. 5D and 5E). Additionally, LEfSe analysis revealed that there were significantly more abundant *Candida albicans*, *Moraxella catarrhalis*, *Candida glabrata*, and *Mycobacterium tuberculosis complex* in the PTB group, *Streptococcus anginosus*, and *Prevotella oris* in the EPTB group, *Haemophilus influenzae*, and Human betaherpesvirus 7 in the non-TB group (Fig. 5F)."

3) **Revised the following paragraph at lines 382–392 in the Discussion section:**

"Our analysis also revealed a significant disruption of the respiratory microbiota in TB patients by mNGS. The reduced alpha diversity in the PTB group indicated a loss of microbial richness and evenness in association with MTB infection. Although the microbial communities were partially intermixed, the greater dispersion in the TB groups suggests increased compositional heterogeneity. The relative abundance of most common species was similar across groups, with *Candida albicans* being a notable differentially abundant species. Some studies have reported a higher prevalence of *Candida albicans* detection in respiratory samples from TB patients [47, 48]. However, differentiating between colonization, contamination, and true co-infection remains challenging. Therefore, whether TB infection truly facilitates opportunistic co-infections or merely alters the microbial landscape without pathogenic consequences is currently unclear. The clinical significance of these microbial shifts requires further investigation."

2. <u>WHO has previously discouraged the use of commercial serological antibody tests, including TB-IgG assays, for diagnosis of active tuberculosis because of inconsistent sensitivity, specificity and potential false-positive results. The rationale for including TB-IgG in the diagnostic comparison should therefore be better justified.</u>

Response:

Yes, we appreciate the reviewer for raising this critical issue. We acknowledge that the WHO 2011 policy statement explicitly discouraged the use of commercial serological tests, including TB-IgG, due to inconsistent performance and the risk of false positives.

However, in China, TB-IgG assays remain widely used in clinical practice, particularly in primary care settings. This is partly because TB-IgG tests are inexpensive, require minimal equipment, and can provide results in 1-2 hours. In contrast, WHO-endorsed methods such as culture (3-8 weeks, BSL-3 requirements) and Xpert MTB/RIF ($10-15 per test) are either time-consuming or costly, making TB-IgG still attractive in resource-limited settings.

Therefore, we did not aim to endorse TB-IgG for routine use. Instead, our objective was to provide a real-world evaluation of the current diagnostic landscape in China, where TB-IgG is still being used, and to generate evidence that could inform policy updates. We have revised the Method to better justify the rationale for including TB-IgG in our comparative study.

Added the following sentences at lines 110–112 in the Method section:

"Although WHO has not recommended the use of serological antibody tests for active TB diagnosis, TB-IgG assays remain in clinical use in some resource-limited regions of China. Therefore, we included this assay to evaluate its real-world performance."

3. <u>In Figure 4A, the combination of Xpert MTB/RIF + culture + AFS already demonstrates very</u>

high specificity (97.4%). The incremental benefit of adding mNGS appears mainly sensitivity-related and should be quantitatively discussed.

Response:

Yes, we thank the reviewer for this precise comment. Based on the data in Figure 4A, adding mNGS increased sensitivity from 51.2% to 70.4%, an absolute increase of 19.2 percentage points. The specificity decreased slightly from 97.4% to 94.2% (a decrease of 3.2 percentage points). The number needed to diagnose (NND) to detect one additional true-positive case by adding mNGS was 5.2, meaning that for every 5-6 patients tested, one additional true-positive case would be identified.

This sensitivity gain is clinically meaningful, as it reduces the risk of missed diagnosis, which is particularly important for a contagious disease like tuberculosis. The modest trade-off in specificity (-3.2%) is acceptable in high-prevalence settings, where false positives can be managed by clinical correlation and follow-up. We have added this quantitative discussion to the revised manuscript (Results and Discussion sections).

1) **Added the following sentence at lines 268–270 in Result section:**

"Adding mNGS increased sensitivity by 19.2 percentage points and decreased specificity by 3.2 percentage points; the number needed to diagnose (NND)[37, 38] to detect one additional true-positive case was 5.2."

2) **Added the following paragraph at lines 358–366 in Discussion section:**

"The incremental diagnostic benefit of adding mNGS was driven primarily by a substantial gain in sensitivity (+19.2 percentage points), with only a modest decrease in specificity (− 3.2 percentage points). This sensitivity improvement is clinically meaningful, as it allows the detection of one additional true-positive case for every 5-6 patients tested (NND = 5.2). In the context of TB control, reducing false negatives outweighs the small increase in false positives, especially when positive mNGS results can be corroborated by clinical and imaging findings. Therefore, a sequential mNGS testing strategy is a powerful approach to improve TB diagnosis, particularly for challenging cases with negative results from other TB assays due to low bacterial load or atypical presentation."

4. The relatively high prevalence of tumors among the study patients, particularly in a cohort of suspected TB cases, warrants further explanation, as malignancy-associated immunosuppression can strongly influence immune parameters, IGRA performance, microbial colonization, opportunistic infections findings, thereby potentially affecting diagnostic interpretation.

Response:

Yes, we thank the reviewer for raising this important concern regarding the relatively high prevalence of tumors in our study cohort and its potential impact on diagnostic interpretation.

In our total cohort of 3,757 patients with suspected tuberculosis, the overall prevalence of malignancy was 15.3% (575/3,757). The highest proportion was observed in the non-TB group (57.7%, 332/575), then EPTB group (30.6%, 176/575), and PTB group (11.7%, 67/575). This reflects the nature of our hospital as a large tertiary referral center, where many patients are referred with undiagnosed pulmonary nodules, mediastinal lymphadenopathy, or unexplained fever, conditions for which both tuberculosis and malignancy are leading differential diagnoses. Therefore, the inclusion of a substantial number of non-TB patients with malignancies is a real-world consequence of diagnostic challenges in such a referral population. Based on the

reviewer's questions, we have conducted further analysis and included it in the supplementary diagram. The specific explanation is as follows. Key evidence that tumor status does not bias our main conclusions: Two independent lines of analysis demonstrate that malignancy-associated immunosuppression did not materially affect the diagnostic performance comparisons.

(1) Correlation heatmap (Figure S1). The heatmap shows a significant negative correlation between TB infection and tumor status (blue, three stars, P < 0.001). This indicates that tuberculosis and malignancy tend to be mutually exclusive in our cohort. Because the vast majority of TB-positive patients, especially those with pulmonary TB, which was the primary focus of our diagnostic sensitivity analysis, were free of malignancy, any potential confounding by tumor-associated immunosuppression on sensitivity estimates is minimal.

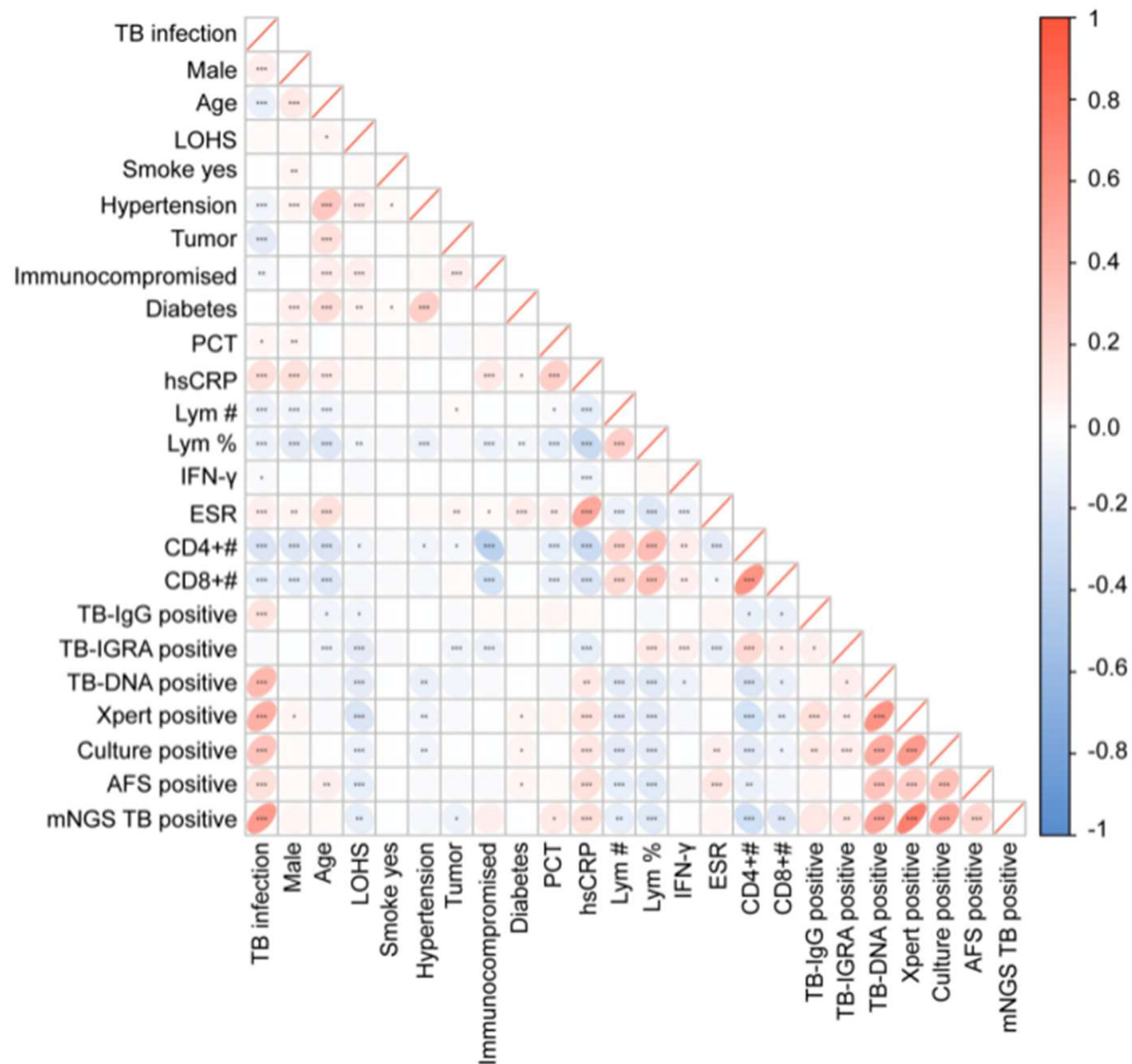

Fig.S1 Spearman correlations between TB infection and characteristics of patients, * *P* < 0.05, ** *P* < 0.01, * *P* < 0.001.**

(2) Stratified logistic regression analysis (Figure S2). We further assessed whether the presence of tumor altered the relative diagnostic performance of mNGS compared with conventional methods (CMTs: culture, Xpert, AFS). The interaction *P* value was > 0.05, which is not significant. Moreover, the odds ratios (OR) for mNGS versus CMTs were similar between the non-tumor subgroup (OR = 1.08, 95% CI: 0.84 – 1.40) and the tumor subgroup (OR = 0.91, 95% CI: 0.53 – 1.56). This directly demonstrates that tumor status did not significantly modify the comparative accuracy of mNGS.

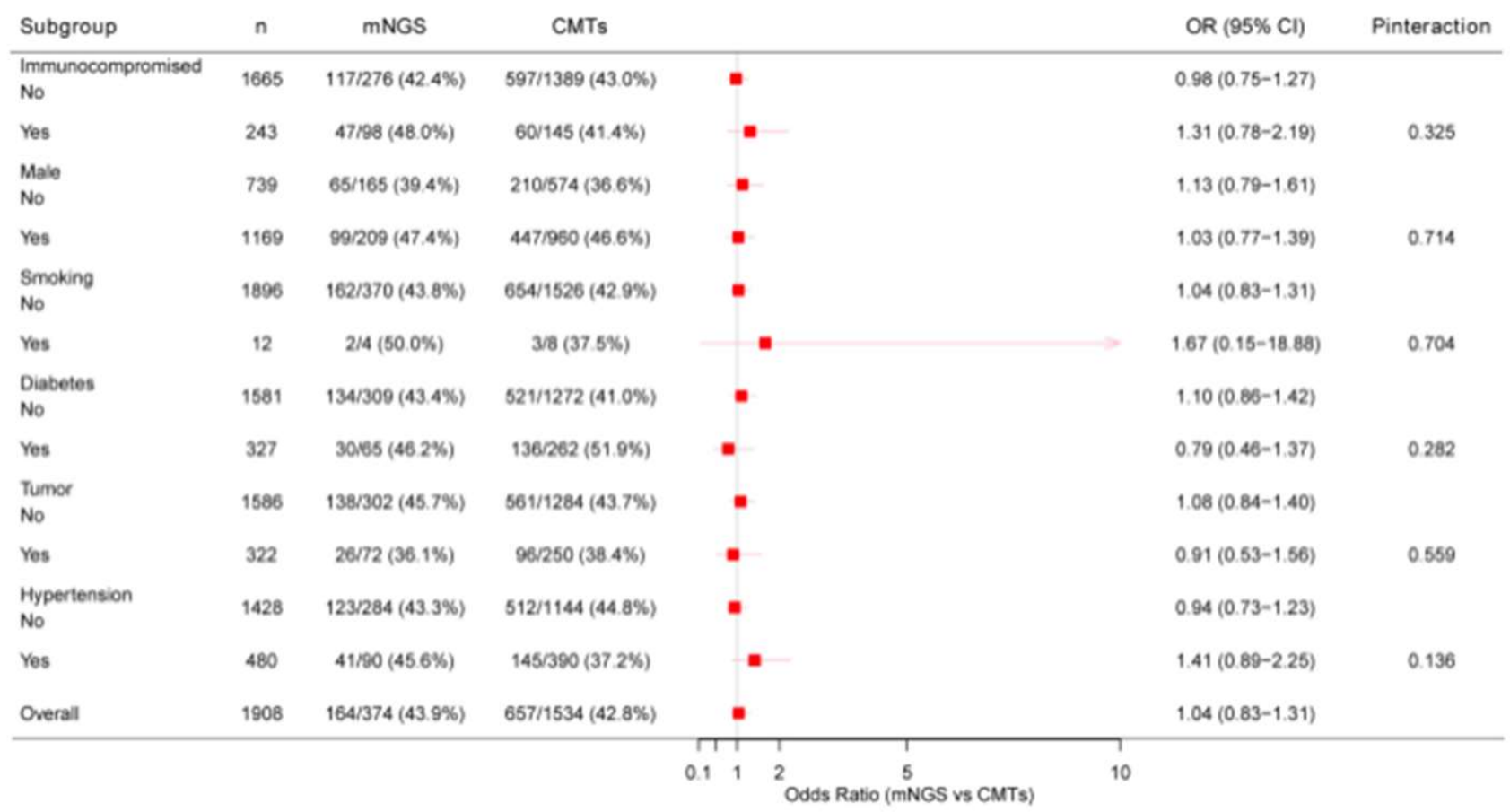

Fig.S3 Subgroup analysis for the proportion of clinical improvements comparing pathogen detection rates between mNGS and CMTs. Pinteraction values, the interaction between the mNGS and subgroup parameters, were reported as Pinteraction values using a logistic model.

The correlation heatmap shows a significant negative correlation between TB and tumor, indicating that TB-positive patients are less likely to have malignancy. Furthermore, stratified logistic regression demonstrated no interaction between tumor status and mNGS performance (P>0.05). These lines of evidence collectively suggest that malignancy-associated immunosuppression did not materially bias our main findings.

Conclusion and limitation: Taken together, the negative correlation ensures that TB-positive patients were largely unaffected by malignancy, and the lack of interaction confirms that even among patients with tumors, mNGS performed comparably relative to conventional methods. Therefore, while we acknowledge that malignancy-associated immunosuppression could theoretically influence immune parameters and IGRA performance, our data indicate that it did not materially bias the core findings of this study. We have added a brief discussion of this point in the Limitations subsection of the revised manuscript.

1) **Added the following sentence at lines 197–198 in the Method section:**

“The spearman correlation analysis was conducted by R package “corrplot” .”

2) **Added the following sentence at lines 214–217 in the Result section:**

“Spearman correlation analyses also showed significant positive correlations between TB infection and male, PCT, hsCRP, and ESR. Significant negative correlations between TB infection and the following variables: age, hypertension, and tumors (Fig. S1). ”

3) **Added the following paragraph at lines 274–280 in the Result section:**

“To further evaluate the diagnostic performance of mNGS compared to Xpert combined with Culture, and AFS (referred as CMTs) across different clinical subgroups, a multilevel logistic regression analysis was conducted. Notably, tests for interaction across all subgroups were not statistically significant (all Pinteraction > 0.05), indicating that the superior detection performance of mNGS over conventional other methods remained consistent among patients with different immune statuses, sexes, smoking histories, diabetic statuses, malignancies, and hypertension conditions, suggesting good generalizability (Fig. S3).”

4) **Added the following sentence at lines 329-330 in the Discussion section:**

"which were consistent with our spearman correlation analyses and the previous reports by WHO[2] and other groups[39-41]. "

5) **Added the following sentence at lines 343-346 in the Discussion section:**

" Furthermore, subgroup analyses showed that the diagnostic performance of mNGS was comparable to that of conventional methods across patients with varying immune statuses, sexes, smoking histories, diabetic statuses, malignancies, and hypertension, indicating consistent generalizability."

5. Minor language revisions are recommended throughout the manuscript to improve grammar, readability, and consistency of terminology. Examples include:
(i) Line 62: "lading" should be corrected to "leading"
(ii) Line 131: The term "wall-breaking procedure" is non-standard and should be replaced with a more appropriate scientific term
(iii) Lines 145 and 149: "mNSG" should be corrected to "mNGS"
(iv) Line 264 and Figure 5D: "Morasella catarrhalis" should be corrected to Moraxella catarrhalis
(v) Figure 5D and 5E: Species names should be italicized according to scientific nomenclature conventions
(vi) Line 314: "cultur" should be corrected to "culture"

Response:

Yes, we thank the reviewer for carefully identifying these typographical and terminological issues. We have corrected all of the following errors in the revised manuscript:
Line 62: "lading" → leading
Line 150: "a wall-breaking procedure" → replaced with "cell lysis"
Lines 170, 174, 176 and 178: "mNSG" → mNGS
Line 294 and Figure 5D: "Morasella catarrhalis" → Moraxella catarrhalis (italicized)
Figure 5D and 5E: All species names have been italicized
Line 355: "cultur" → culture
In addition, we have carefully proofread the entire manuscript to correct any other similar typographical or grammatical issues, and we have standardized terminology for consistency. We believe the revised manuscript now meets the required language standards. Thank you again for these constructive suggestions.

Response to Reviewer #3

Major comments:

• Clarify reference standard construction: Provide explicit details on how the composite reference standard was constructed, including adjudication procedures for discordant results.

Response:

Yes, we thank the reviewer for this important methodological suggestion. The final clinical diagnosis documented in medical records served as the composite reference standard, following the *Chinese Clinical Treatment Guide for Tuberculosis (2017 Edition)* and WHO recommendations, integrating microbiological, radiological, clinical, and treatment-response evidence. For discordant results, the final diagnosis was independently determined by two licensed

physicians blinded to the mNGS results; disagreements were resolved by consensus or by consultation with a third senior clinician. Patients with insufficient data for definitive classification were excluded from the primary analysis. In the revised manuscript, we have added a dedicated subsection titled "Composite reference standard and adjudication of discordant results" in the Methods section.

1) **Added the following sentence at lines 116–128 in the Method section:**
"Composite reference standard and adjudication of discordant results
The final clinical diagnosis recorded in the medical records served as the composite reference standard. The diagnosis was based on the Chinese Clinical Treatment Guide for Tuberculosis (2017 Edition) and WHO recommendations, integrating microbiological evidence (positive culture or Xpert MTB/RIF), radiological findings (chest CT), clinical symptoms (cough ≥2 weeks, fever, night sweats, weight loss), treatment response to anti-TB therapy, and exclusion of alternative diagnoses. Importantly, mNGS was performed retrospectively and was not available to the treating physicians at the time of diagnosis, ensuring independence of the reference standard from the index test. For discordant results (e.g., negative microbiological tests but strong radiological and clinical suspicion), the final diagnosis was determined by two licensed physicians (JL and JZ) who were blinded to the mNGS results; disagreements were resolved by consensus or by consultation with a third senior clinician (XYT). Patients with insufficient data for definitive classification were excluded from the primary analysis."

• Statistical power analysis: Include a priori sample size calculations to justify the adequacy of specimen-type-specific cohorts.

Response:
Yes, we thank the reviewer for this important statistical comment. We have carefully examined each specimen-type-specific cohort.

Adequacy of sample sizes: As shown in Supplementary Table S3 (newly added), the BALF subgroup (n=354) provided a sufficient sample size, with a sensitivity 95% confidence interval half-width of 7.7%, which is within the acceptable precision of 10%. However, the other specimen types (CSF, blood, tissue, body fluid, and others) had smaller numbers of positive cases, resulting in wider confidence intervals (half-widths ranging from 22% to 56%). Consequently, the diagnostic performance estimates for these subgroups should be interpreted with caution. we have revised in the Discussion section and added a Supplementary Table S3.

Supplementary Table S3 Specimen-type-specific sample sizes and estimated precision (95% CI half-width) of sensitivity, assuming Se=59% and Sp=100%

Specimen type	pos (n)	neg (n)	Sensitivity 95% CI half-width	Adequacy
BALF	159	195	7.7%	Adequate
CSF	19	45	22.3%	Limited
Blood	5	30	43.5%	Very limited
Tissue	16	10	24.3%	Limited
Body fluid	8	28	34.4%	Limited
Others	3	10	55.9%	Exploratory only

Note: The 95% CI half-widths for sensitivity were calculated using the Wilson score method, assuming a sensitivity of 59% and a specificity of 100% (the observed overall estimates). For specimen types where specificity was 100% in the actual data, the CI half-width for specificity is negligible and not shown. These precision estimates are provided to illustrate the statistical reliability of each subgroup; actual confidence intervals may vary slightly depending on the observed sensitivity in each subgroup.

1) **Added the following sentence at lines 353–354 in the Discussion section:**
"For non-BALF specimen types, further prospective studies with expanded specimen size are needed to achieve more accurate estimates of diagnostic accuracy (Table S3)."

2) **Added the following sentence at lines 419–422 in the Discussion section:**
"Third, the sample sizes for non-BALF specimen types (e.g., CSF, blood, tissue, body fluid) were relatively small, leading to imprecise sensitivity estimates. Therefore, these results are hypothesis-generating and require validation in larger prospective studies (Table S3). "

• Supplementary data: Consider providing detailed supplementary tables with specimen-type-specific performance metrics and stratified cost-effectiveness analyses by TB subtype (PTB vs. EPTB).

Response:

Yes, we thank the reviewer for this constructive suggestion. As suggested, we have now prepared two supplementary tables and figures as recommended.

Specimen-type-specific performance metrics (Table S1 and fig.S2): Following the reviewer's advice, we added detailed supplementary tables with specimen-type-specific performance metrics. For each specimen type, we have calculated sensitivity, specificity, PPV, NPV, AUC and their 95%

confidence intervals. This table directly demonstrates how test performance may vary depending on the sample source. And we have revised the Result and Discussion part.

Table S1 Specimen-type-specific of mNGS performance metrics

Specimen type	Sensitivity	specificity	accuracy	PPV	NPV	AUC
BALF	71.0%[64.7%-76.5%]	100.0%[97.1%-100.0%]	81.6%[77.3%-85.3%]	100.0%[97.6%-100.0%]	66.7%[59.8%-72.9%]	0.855(0.816, 0.894)
CSF	36.5%[24.8%-50.1%]	100.0%[75.7%-100.0%]	48.4%[36.6%-60.4%]	100.0%[83.2%-100.0%]	26.7%[16.0%-41.0%]	0.683(0.544, 0.822)
Blood	25%[11.2%- 46.9%]	100.0%[79.6%-100.0%]	57.1%[40.9%-72.0%]	100%[56.6%-100.0%]	50.0%[33.2%-66.8%]	0.625(0.4406, 0.8094)
Tissue	72.7%[51.8%-86.9%]	100.0%[51.0%-100.0%]	76.9%[58.0%-89.0%]	100.0%[80.6%-100.0%]	40.0%[16.8%-68.7%]	0.864(0.7212, 1.000)
Body fluid	27.6%[14.7% -45.7%]	100.0%[64.6%-100.0%"]	41.7%[27.1%-57.8%]	100.0%[67.6%-100.0%]	25.5%[12.7%-43.4%]	0.638(0.4378, 0.8380)
Others	33.3%[12.1%-64.6%]	100.0%[51.0%-100.0%]	53.8%[29.1%-76.8%]	100.0%[43.9%-100.0%]	40.0%[16.8%-68.7%]	0.667(0.3652, 0.9682)

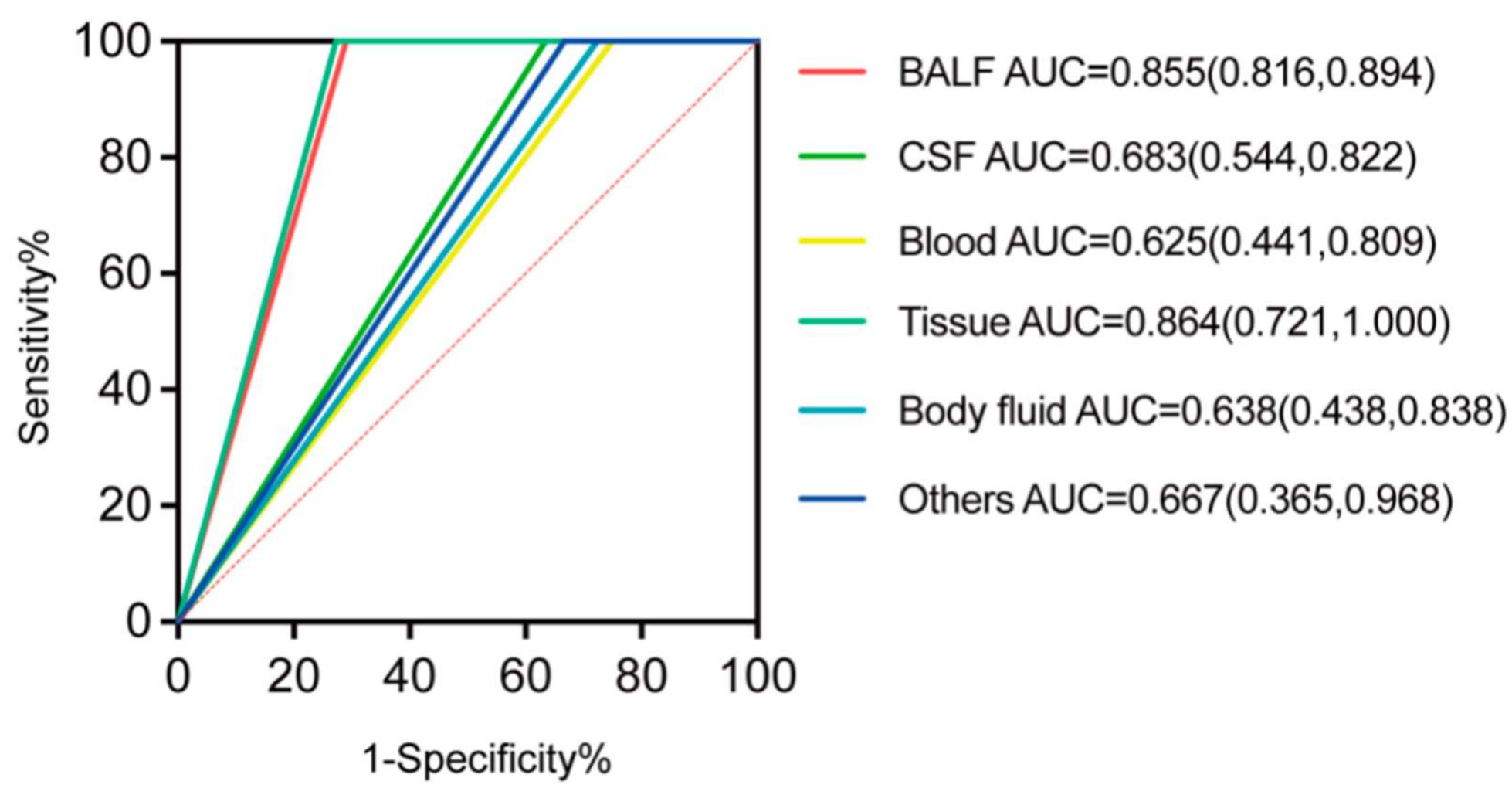

Fig.S2 ROC curve of specimen-type-specific of mNGS for discrimination between TB and non-TB infection by negative or positive.

1) **Revised the following sentence at lines 254–260 in the Result section:**

"We compared the diagnostic performances of mNGS in all specimen types (Table S1) and showed the top three types in figure 3. The specificities and PPVs were all 100% as overall types, while both tissue (72.7%) and BALF (71.0%) had superiors sensitivity compared to CSF (36.5%). Especially, mNGS possessed the highest accuracy (81.6%), NPV (66.7%), and AUC (0.855; 95% CI: 0.816-0.894) in BALF among all types (Fig. 3B, Fig. S2, and Table S1), indicating BALF is the best specimen type for TB diagnosis with mNGS. "

2) **Revised the following sentence at lines 348–351 in the Discussion section:**

"Both specificity and PPV were 100% for all specimen types. Its sensitivity was significantly higher in BALF (71.0%) and tissue (72.7%) compared to CSF (36.5%) and other specimen types. And the accuracy (81.6%), NPV (66.7%) and AUC (0.855; 95% CI: 0.816-0.894) in BALF specimens were much higher than those in other specimen types. "

Stratified cost-effectiveness analysis by TB subtype (Table S2, Fig.S4 and Fig.S5): We have conducted a stratified cost-effectiveness analysis separately for PTB and EPTB patients, using the incremental cost-effectiveness ratio (ICER) as the primary outcome measure. The ICER was calculated as the ratio of the incremental cost of the diagnostic strategy (e.g., Xpert+culture+AFS+mNGS vs. Xpert+culture+AFS) to the incremental effect (additional true-positive cases detected). And we have revised the Result and Discussion part.

Table S2 Total cost analyses of Xpert+culture+AFS, and Xpert+culture+AFS+mNGS, for PTB infection and EPTB infection

Test	N	Mean(SD) ¥	Median(IQR), ¥	Adjusted mean* (95%CI) ¥	Adjusted mean difference(95% CI) (mNGS vs Other3)	Adjusted relative ratio
PTB						
Xpert+culture+AFS	595	24567 (63996)	14239 (10477, 19133)	22851 (19772, 25930)	3192 (64, 6320), p<0.001	1.14 (1.00, 1.29), p=0.042
Xpert+culture+AFS+mNGS	170	25117 (29939)	18647 (14890, 22560)	26043 (22343, 29743)		
EPTB						
Xpert+culture+AFS	263	25715 (27535)	18434 (12705, 26159)	25628 (22928, 28329)	13987 (7043, 20931), p<0.001	1.55 (1.28, 1.37), p<0.001
Xpert+culture+AFS+mNGS	53	39623 (27260)	27793 (19370, 57158)	39615 (33182, 46048)		

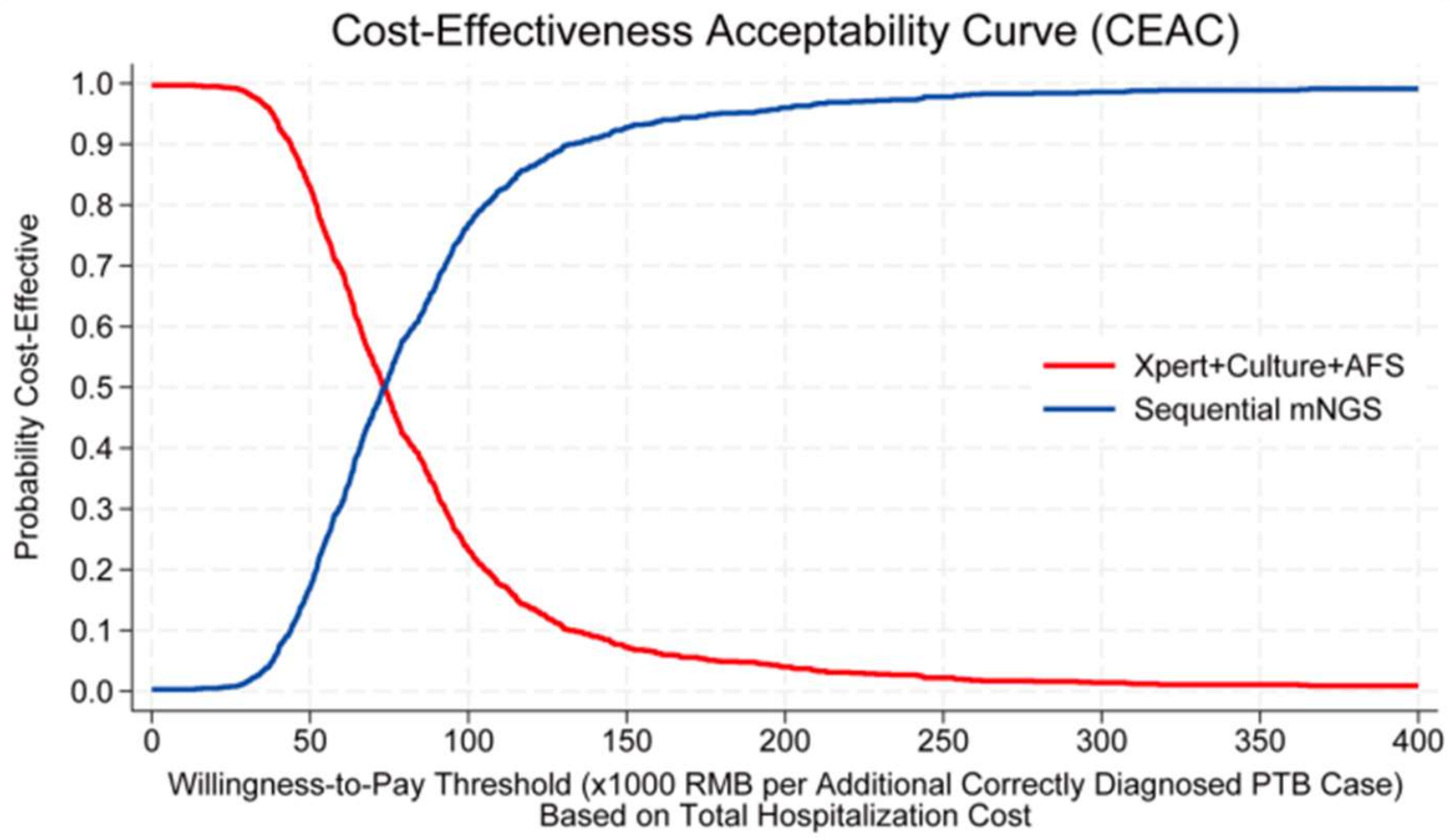

Fig.S4 Cost-effectiveness acceptability curves for mNGS method of the PTB diagnosis.

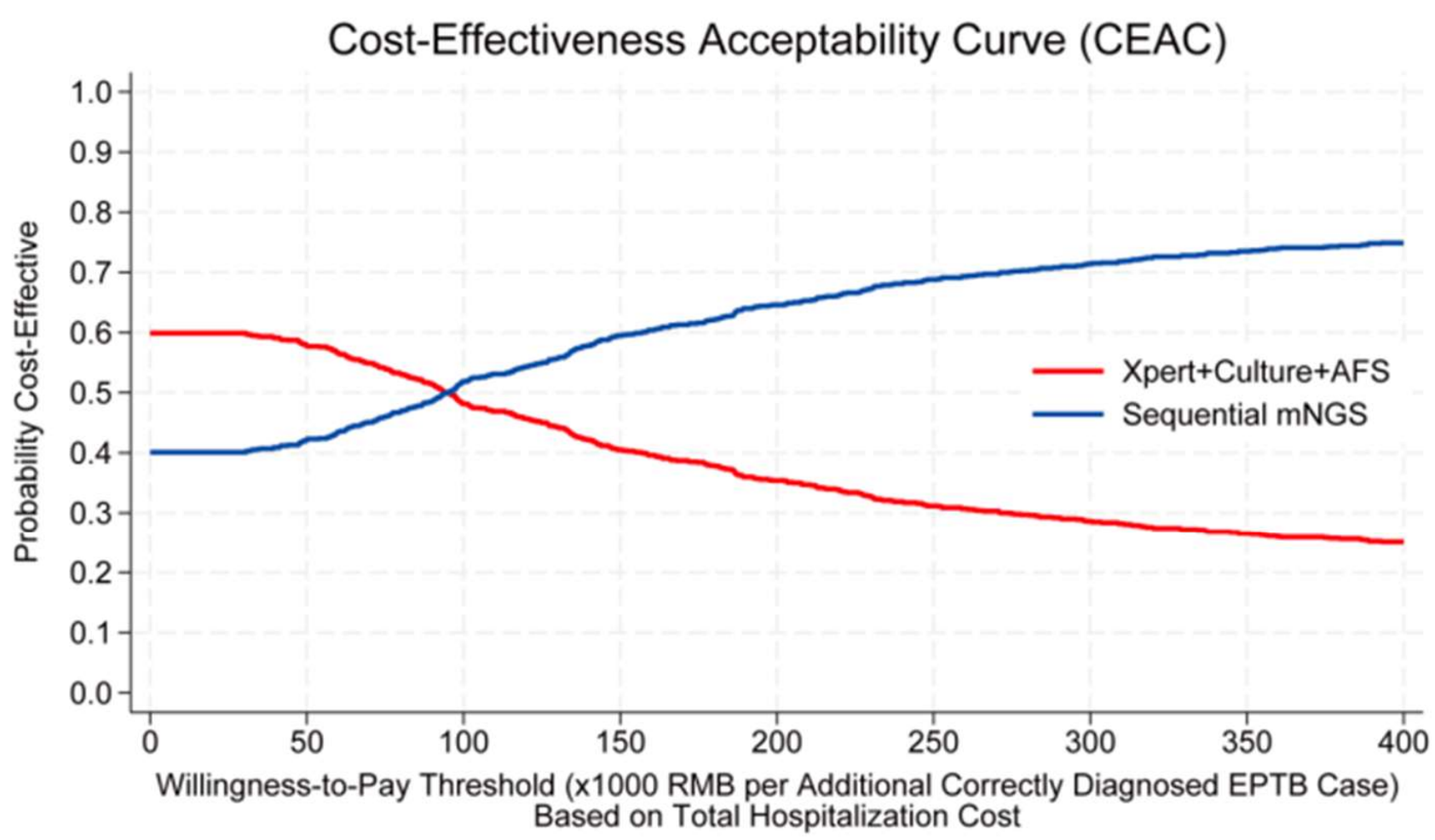

Fig.S5 Cost-effectiveness acceptability curves for mNGS method of the EPTB diagnosis.

1) **Added the following sentence at lines 308–309 and 321-324 in the Result section:**

"Consistent results showing the same trend were also observed in TB subtype PTB and EPTB (Table S2)."

"Sequential mNGS becomes the optimal cost-effective choice, with near-certain probabilities of >90% and >70% at WTP thresholds of approximately 150,000 RMB (for PTB) and 300,000 RMB (for EPTB) per additional correctly diagnosed case, respectively (Fig. S4 and Fig. S5). "

2) **Added the following sentence at lines 403–405 in the Discussion section:**

"Sequential mNGS is more cost-effective at higher WTP thresholds, with a lower threshold and a more pronounced advantage for PTB. "

• Discussion of contamination: Expand the discussion with specific mNGS contamination rates observed in this cohort and describe decontamination protocols employed.

Response:

Yes, we thank the reviewer for raising this critical point regarding mNGS contamination. In this retrospective cohort study, no mNGS false-positive results attributable to contamination were observed in the non-TB group. This was achieved by including: (1) inclusion of negative/positive controls per batch; (2) removal of reads belonging to a predefined list of common environmental background genera; (3) physical separation of laboratory areas; and (4) retesting of samples when contamination is suspected by the reviewing physician. We have added this and revised manuscript in Method section.

1) **Added the following sentence at lines 160–165 in the Method section:**

"To minimize contamination, strict physical separation of pre- and post-PCR areas with a unidirectional workflow was enforced. Each sequencing batch included a negative control (extraction blank) and a positive control (mock community). Reads belonging to a predefined list of common environmental background genera were removed from the final report. If contamination was suspected by the reviewing physician based on control results or abnormal read patterns, the sample was retested."

2) **Added the following sentence at lines 372–374 in the Discussion section:**

"Additionally, our stringent contamination control protocols (physical separation, batch controls, background read removal, and retesting upon suspicion) effectively minimized false positives, strengthening confidence in the mNGS results presented."

• External validation: Briefly discuss the need for external validation in diverse healthcare settings to confirm the proposed tiered diagnostic approach.

Response:

Yes, we thank the reviewer for this important perspective. As suggested, we have added a brief discussion in the limitations subsection of the Discussion section, explicitly acknowledging that our findings are derived from a single-center retrospective cohort and may not be directly generalizable to other settings. We further note that external validation in multi-center, prospective studies across diverse healthcare environments will be essential to confirm the clinical utility of the proposed tiered diagnostic approach. We have revised the manuscript in the Discussion section.

Added the following sentence at lines 422–426 in the Discussion section:

"Fourth, because our proposed tiered diagnostic approach was evaluated in a single tertiary referral center, its generalizability to other healthcare settings (e.g., primary care facilities, district hospitals, or regions with different TB epidemiology) remains uncertain. External validation in diverse clinical and geographic settings is needed to confirm the robustness and reproducibility of our conclusions before widespread implementation."

Re: Spectrum00970-26R1 (**Metagenomic Next-Generation Sequencing for Tuberculosis Diagnosis: Enhanced Performance and Cost-Effectiveness**)

Dear Dr. Li Jiang:

Your manuscript has been accepted, and I am forwarding it to the ASM production staff for publication. Your paper will first be checked to make sure all elements meet the technical requirements. ASM staff will contact you if anything needs to be revised before copyediting and production can begin. Otherwise, you will be notified when your proofs are ready to be viewed.

Data Availability: ASM policy requires that data be available to the public upon online posting of the article, so please verify all links to sequence records, if present, and make sure that each number retrieves the full record of the data. If a new accession number is not linked or a link is broken, provide production staff with the correct URL for the record. If the accession numbers for new data are not publicly accessible before the expected online posting of the article, publication may be delayed; please contact ASM production staff immediately with the expected release date.

Publication Fees: For information on publication fees and which article types have charges, please visit our website. We have partnered with Copyright Clearance Center (CCC) to collect author charges. If fees apply to your paper, you will receive a message from no-reply@copyright.com with further instructions. For questions related to paying charges through RightsLink, please contact CCC at ASM_Support@copyright.com or toll free at +1-877-622-5543. CCC makes every attempt to respond to all emails within 24 hours.

ASM Membership: Corresponding authors may join or renew ASM membership to obtain discounts on publication fees. Need to upgrade your membership level? Please contact Customer Service at Service@asmusa.org.

Embargo Policy: A press release may be issued as soon as the manuscript is posted on the Spectrum Latest Articles webpage. The corresponding author will receive an email with the subject line "ASM Journals Author Services Notification" when the article is available online.

The ASM Journals program strives for constant improvement in our submission and publication process. Please tell us how we can improve your experience by taking this quick Author Survey.

Thank you for submitting your paper to Spectrum.

Sincerely,
Benjamin Liu
Editor
Microbiology Spectrum
