# Supplementary material for: Metagenomic next-generation sequencing for tuberculosis diagnosis: enhanced performance and cost-effectiveness
Source: Microbiol Spectr. 2026 Jul 29;14(9):e00970-26. doi: 10.1128/spectrum.00970-26 (PMC13532181; doi:10.1128/spectrum.00970-26)
Supplement: Supplementary file 2 [file spectrum.00970-26-s0006.docx]

**Supplementary figures and tables**

**Fig.S1 Spearman correlations between TB infection and characteristics of patients, * *P* < 0.05, ** *P* < 0.01, *** *P* < 0.001.**

**Fig.S2 ROC curve of specimen-type-specific of mNGS for discrimination between TB and non-TB infection by negative or positive.**

**Fig.S3 Subgroup analysis for the proportion of clinical improvements comparing pathogen detection rates between mNGS and CMTs. Pinteraction values, the interaction between the mNGS and subgroup parameters, were reported as Pinteraction values using a logistic model.**

**Fig.S4 Cost-effectiveness acceptability curves for mNGS method of the PTB diagnosis.**

**Fig.S5 Cost-effectiveness acceptability curves for mNGS method of the EPTB diagnosis.**

Table S1 Specimen‑type‑specific of mNGS performance metrics

| Specimen type | Sensitivity | specificity | accuracy | PPV | NPV | AUC |
| --- | --- | --- | --- | --- | --- | --- |
| BALF | 71.0%[64.7%-76.5%] | 100.0%[97.1%-100.0%] | 81.6%[77.3%-85.3%] | 100.0%[97.6%-100.0%] | 66.7%[59.8%-72.9%] | 0.855(0.816, 0.894) |
| CSF | 36.5%[24.8%-50.1%] | 100.0%[75.7%-100.0%] | 48.4%[36.6%-60.4%] | 100.0%[83.2%-100.0%] | 26.7%[16.0%-41.0%] | 0.683(0.544, 0.822) |
| Blood | 25%[11.2%- 46.9%] | 100.0%[79.6%-100.0%] | 57.1%[40.9%-72.0%] | 100%[56.6%-100.0%] | 50.0%[33.2%-66.8%] | 0.625(0.4406, 0.8094) |
| Tissue | 72.7%[51.8%-86.9%] | 100.0%[51.0%-100.0%] | 76.9%[58.0%-89.0%] | 100.0%[80.6%-100.0%] | 40.0%[16.8%-68.7%] | 0.864(0.7212, 1.000) |
| Body fluid | 27.6%[14.7% -45.7%] | 100.0%[64.6%-100.0%"] | 41.7%[27.1%-57.8%] | 100.0%[67.6%-100.0%] | 25.5%[12.7%-43.4%] | 0.638(0.4378, 0.8380) |
| Others | 33.3%[12.1%-64.6%] | 100.0%[51.0%-100.0%] | 53.8%[29.1%-76.8%] | 100.0%[43.9%-100.0%] | 40.0%[16.8%-68.7%] | 0.667(0.3652, 0.9682) |

Table S2 Total cost analyses of Xpert+culture+AFS, and Xpert+culture+AFS+mNGS, for PTB infection and EPTB infection

| Test | N | Mean(SD) ¥ | Median(IQR),  ¥ | Adjusted mean*  (95%CI) ¥ | Adjusted mean difference(95%CI) (mNGS vs Other3) | Adjusted relative ratio |
| --- | --- | --- | --- | --- | --- | --- |
| PTB | | | | | | |
| Xpert+culture+AFS | 595 | 24567 (63996) | 14239 (10477, 19133) | 22851 (19772, 25930) | 3192 (64, 6320), *P*<0.001 | 1.14 (1.00, 1.29), *P*=0.042 |
| Xpert+culture+AFS+mNGS | 170 | 25117 (29939) | 18647 (14890, 22560) | 26043 (22343, 29743) |  |  |
| EPTB | | | | | | |
| Xpert+culture+AFS | 263 | 25715 (27535) | 18434 (12705, 26159) | 25628 (22928, 28329) | 13987 (7043, 20931), *P*<0.001 | 1.55 (1.28, 1.37), *P*<0.001 |
| Xpert+culture+AFS+mNGS | 53 | 39623 (27260) | 27793 (19370, 57158) | 39615 (33182, 46048) |  |  |

*We used a generalized linear model (GLM) with Gamma family and log link, adjusting for demographic (age, sex, smoking), comorbidities (diabetes, tumor, hypertension, immunosuppression), surgery status, and inflammatory marker categories. Cluster-robust standard errors were used at the inpatient-ID level

SD: Standard Deviation

IQR: Interquartile Range (25th percentile, 75th percentile)

CI: Confidence Interval

Table S3 Specimen‑type‑specific sample sizes and estimated precision (95% CI half‑width) of sensitivity, assuming Se=59% and Sp=100%

| Specimen type | pos (n) | neg (n) | Sensitivity 95% CI half‑width | Adequacy |
| --- | --- | --- | --- | --- |
| BALF | 159 | 195 | 7.7% | Adequate |
| CSF | 19 | 45 | 22.3% | Limited |
| Blood | 5 | 30 | 43.5% | Very limited |
| Tissue | 16 | 10 | 24.3% | Limited |
| Body fluid | 8 | 28 | 34.4% | Limited |
| Others | 3 | 10 | 55.9% | Exploratory only |

Note: The 95% CI half‑widths for sensitivity were calculated using the Wilson score method, assuming a sensitivity of 59% and a specificity of 100% (the observed overall estimates). For specimen types where specificity was 100% in the actual data, the CI half‑width for specificity is negligible and not shown. These precision estimates are provided to illustrate the statistical reliability of each subgroup; actual confidence intervals may vary slightly depending on the observed sensitivity in each subgroup.
